# Broadening Hard‐Magnet Discovery Beyond Symmetry Constraints via Unified Effective Anisotropy

**DOI:** 10.1002/advs.202524393

**Published:** 2026-09-08

**Authors:** Hojae Kim, Hyeondeok Shin, Kyungju Nam, Seunghyo Noh, Donghwi Kim, Yousung Jung

**Affiliations:** ^1^ Department of Chemical and Biological Engineering and Institute of Chemical Processes Seoul National University Seoul Republic of Korea; ^2^ Computational Science Division Argonne National Laboratory Lemont Illinois USA; ^3^ Materials Research & Engineering Center Hyundai Motor Company Uiwang‐si Gyeonggi‐do Republic of Korea; ^4^ Institute of Engineering Research Seoul National University Seoul Republic of Korea

**Keywords:** density functional theory, generative models, hard magnetic materials, magnetocrystalline anisotropy, materials informatics

## Abstract

Machine‐learning‐driven discovery of rare‐earth‐lean hard magnets has advanced rapidly, yet existing pipelines have remained constrained either to a fixed composition family or to a single crystal‐system class, leaving most compositional and structural space unexplored. We address both limitations by introducing a unified effective anisotropy constant, K_eff_, defined consistently across all seven crystal systems, equal to the conventional K_1_ in uniaxial cases and—as established here by symmetry‐based derivation and density functional theory calculations—designed to approximate the minimum magnetization‐reversal barrier that governs the hardness parameter κ in orthorhombic, monoclinic, and triclinic systems, with symmetry‐dependent accuracy quantified by angular DFT sampling. With this descriptor, we assemble a magnetocrystalline‐anisotropy database and train a two‐stage classification‐regression pipeline that screens structures without symmetry prefiltering. Applied to Materials Project entries, it recovers established hard magnets and uncovers rare‐earth‐free κ > 1 candidates in orthorhombic and monoclinic classes—beyond most previous symmetry‐restricted screens. Complementing this search, a fine‐tuned diffusion model proposes five DFT‐confirmed rare‐earth‐free candidates, four absent from the Materials Project: refinements of a known Pt hard‐magnet chemistry plus the noble‐metal‐free TaFe_3_, three of them phonon‐stable. These results show that a physically unified anisotropy descriptor can substantially broaden data‐driven hard‐magnet discovery using established machine‐learning and generative workflows.

## Graphical abstract

A unified effective‐anisotropy descriptor (*K*_eff_) extends hard‐magnet screening across all seven crystal systems, beyond the uniaxial restriction of conventional searches. Machine‐learning screening of 9320 known ferromagnets and diffusion‐model generation together yield 38 rare‐earth‐free or ‐lean candidates with DFT‐validated magnetic hardness (κ > 1), including the compositionally distinct, noble‐metal‐free TaFe_3_.

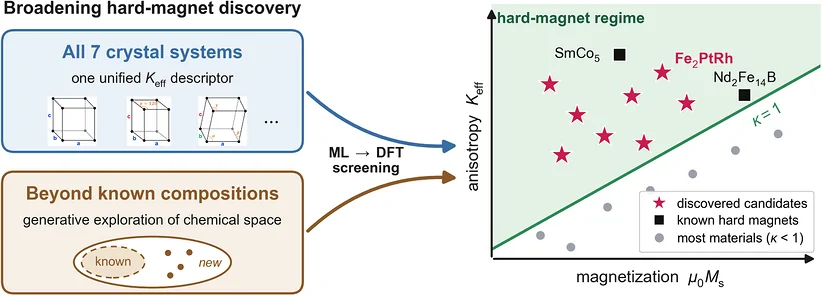

## 1 Introduction

Hard magnets, ferromagnets that retain their magnetization through large magnetocrystalline anisotropy (MCA), are essential to high‐efficiency electrified technologies, from electric‐vehicle motors to wind‐turbine generators and compact consumer electronics. High‐anisotropy magnetic materials are equally central to ultrahigh‐density information storage: the rapid expansion of data‐center capacity has renewed demand for heat‐assisted magnetic recording (HAMR) media, in which nanometer‐scale grains must retain their magnetization against thermal fluctuations, placing a premium on materials that combine large MCA with adequate magnetization. Over the past decade [1, 2], their performance and supply chain stability have emerged as critical global challenges. Since state‐of‐the‐art magnets rely heavily on scarce and expensive rare‐earth elements [3, 4], extensive research has focused on discovering rare‐earth‐lean or rare‐earth‐free alternatives that maintain high coercivity and magnetization. While traditional trial‐and‐error discovery is prohibitively slow, and even high‐throughput computation is hindered by the cost of evaluating magnetic properties, machine‐learning (ML) has begun to bridge this gap. ML models now enable rapid property prediction from structure and chemistry [5], while generative frameworks such as DiffCSP and MatterGen further extend these capabilities by proposing novel candidate structures beyond established chemical spaces [6, 7].

For practical applications, “hard‐magnet” behavior is characterized by high saturation magnetization (M_s_) and high coercivity (H_c_). However, direct prediction of coercivity is notoriously difficult, as it depends on extrinsic microstructure‐sensitive factors such as defects, grain boundaries, and domain‐wall dynamics. While micromagnetic simulations based on the Landau–Lifshitz–Gilbert equation can model these processes [8], they are computationally prohibitive for large‐scale screening and often overestimate experimental values, a phenomenon known as the Brown paradox [9]. To maintain a first‐principles foundation while bypassing these extrinsic complexities, MCA energy (E_MCA_) is widely adopted as an intrinsic, processing‐independent proxy for coercivity. Computed from spin–orbit coupling (SOC) energy differences between crystallographic axes [10, 11], E_MCA_ can be normalized by volume to yield K_1_, the primary intrinsic anisotropy constant that governs the coercivity scale of a material. Recent work has further identified universal electronic‐structure relationships governing these intrinsic magnetic properties across diverse permanent‐magnet families [12]. As an intrinsic single‐crystal quantity, K_1_ provides a processing‐independent upper bound on achievable coercivity; actual coercive fields in polycrystalline magnets depend additionally on microstructural factors—grain size, domain‐wall pinning, demagnetization geometry—and typically fall well below this limit [9]. The candidates identified here therefore represent high intrinsic anisotropy potential, with practical coercivity subject to further microstructural optimization.

Importantly, M_s_ and K vary largely independently. While a high M_s_ is desirable for the energy product of bulk permanent magnets, the defining requirement of a hard magnet is not a high value of either quantity in isolation but a sufficiently large anisotropy relative to the magnetization, so that the material resists self‐demagnetization. A large M_s_ increases the internal demagnetizing field, which tends to destabilize the magnetic state; consequently, a sufficiently large K_1_ is required to counteract this effect. This critical balance is effectively captured by the dimensionless magnetic hardness parameter κ = √(K_eff_/µ_0_M_s_^2^), which measures the anisotropy strength relative to the magnetization. A material is generally classified as a hard magnet when κ > 1, indicating that the MCA is sufficiently large to resist self‐demagnetization and sustain magnetization, regardless of the sample geometry [13].

However, the physical subtlety of MCA presents a significant challenge for predictive modeling. Historically, the difficulty of capturing the intricate interplay between crystal structure and magnetic anisotropy has restricted computational screening to narrower, homogeneous domains, such as specific chemical families [10, 14, 15, 16, 17] or limited crystallographic systems [18, 19, 20], thereby risking systematic bias and overlooked discoveries. For instance, seminal high‐throughput studies have systematically explored ternary Fe‐Co‐X (X = B, C, N, P, Si) systems [11, 21, 22, 23, 24] by leveraging the chemical intuition that Fe provides high magnetization while Co enhances anisotropy. While highly effective, this intuition‐driven approach is inherently confined to a predefined chemical space, hindering the exploration of unconventional regions.

We substantially alleviate this long‐standing composition–symmetry limitation by introducing a unified descriptor that can be consistently applied across all seven crystal systems. The principal contribution of this work is not a new machine‐learning architecture, but a physically unified anisotropy descriptor that enables a single screening framework across crystal symmetries. K_eff_ recovers the conventional K_1_ for uniaxial systems and, as we establish through symmetry‐based derivation and dedicated density functional theory (DFT) validation, provides a physically motivated approximation to the minimum magnetization‐reversal barrier in low‐symmetry systems, with accuracy that depends on crystal symmetry as quantified in Section 3.1. This single physical descriptor enables a two‐stage classification–regression pipeline that screens crystal structures across the full periodic table and all crystal classes without imposing composition or symmetry pre‐filters. We pair two complementary searches: a screen of experimentally reported structures that grounds the database track in known chemistries, and a fine‐tuned diffusion generative model that proposes candidates not present in known databases, spanning compositions absent from the database‐derived hard‐magnet set. The resulting workflow recovers a wide array of established hard magnets while yielding unexplored κ > 1 candidates spanning low‐symmetry crystal classes that prior pipelines have not generally explored.

## 2 Methods

### 2.1 Construction of the MCA DFT Database

**FIGURE 1:**
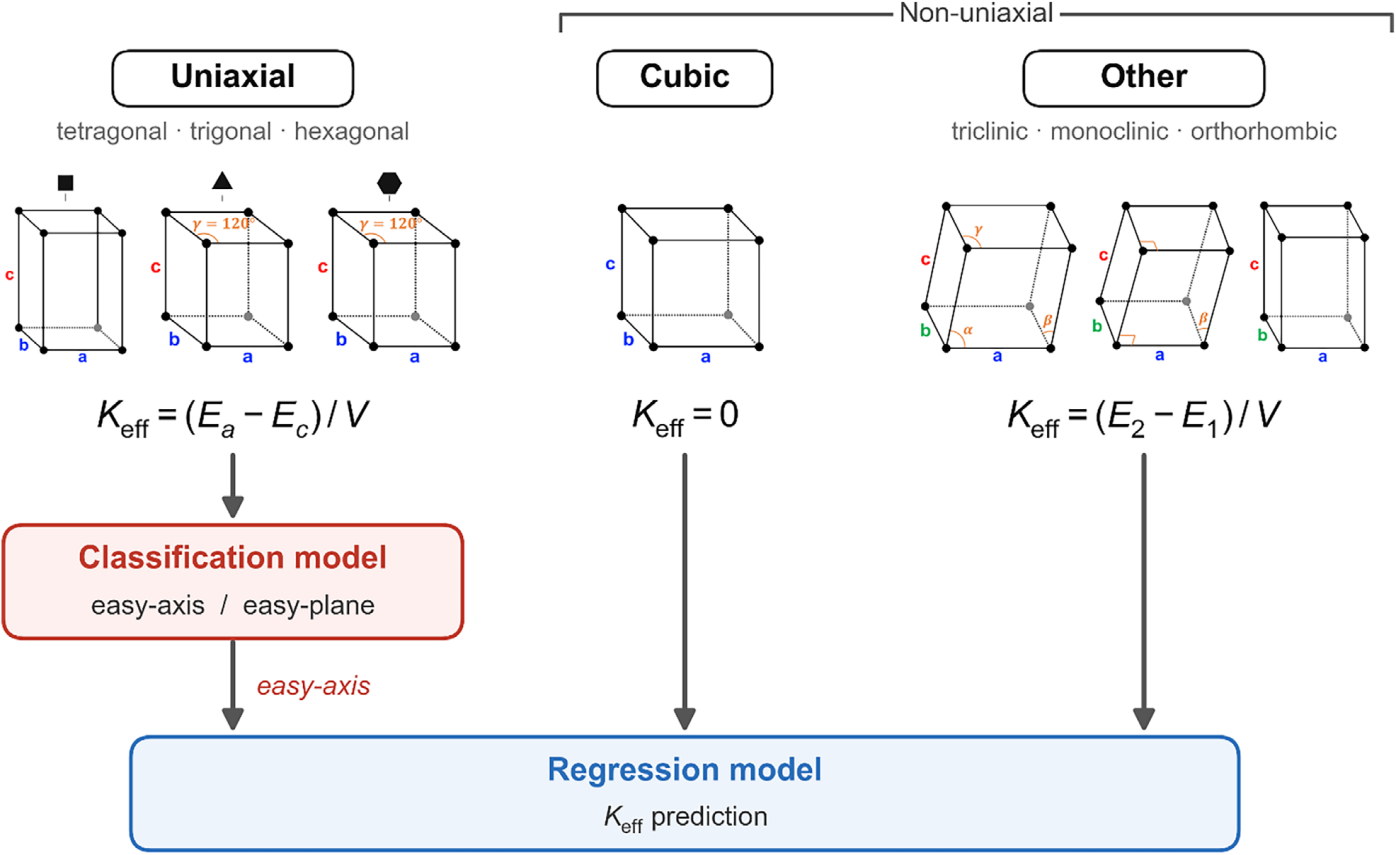
Unified effective‐anisotropy (K_eff_) descriptor and two‐stage learning scheme. Uniaxial systems undergo easy‐axis/easy‐plane classification before K_eff_ regression, whereas non‐uniaxial systems enter the regression model directly.

To define a symmetry‐consistent MCA descriptor applicable to all crystal systems, we followed the scheme summarized in Figure 1. For uniaxial structures (tetragonal, trigonal, and hexagonal), the effective anisotropy constant was computed as

$$K_{eff}=\frac{E_{a}-E_{c}}{V}$$

where c denotes the principal axis. This expression corresponds to the first anisotropy constant K_1_ at leading (second) order, as higher‐order contributions in these systems are typically small [25]. For lower‐symmetry structures (triclinic, monoclinic, and orthorhombic), where a unique K_1_ is not formally defined [25], we evaluated K_eff_ using the energy difference between the lowest (E_1_) and second‐lowest (E_2_) spin–orbit–coupled orientations among the three conventional crystallographic axes:

$$K_{eff}=\frac{E_{2}-E_{1}}{V}$$

For cubic systems, the three orientations are energetically equivalent under SOC, yielding

$$K_{eff}=0$$

consistent with the absence of a leading uniaxial anisotropy term [25]. Structures containing f‐elements were computed using element‐specific high‐accuracy settings. All first‐principles calculations were performed with VASP [26, 27], SOC calculations used an energy cutoff of 520 eV and k‐point densities exceeding 50 k‐points/Å^−^^3^; the anisotropy targeted by K_eff_ is the reversal barrier of the net magnetization and was therefore evaluated on the high‐spin ferromagnetic configuration. For each generated candidate carried to final DFT validation, the status of this configuration as the true collinear ground state was established by explicit enumeration rather than assumed; for MP‐derived candidates, the database magnetic labels were treated as screening descriptors and subsequently checked against the available literature (Section 3.3). Detailed settings such as composition‐dependent parameters are provided in the Supporting Information (SI) Section S1.

To build an MCA‐labeled dataset matched to the deployment domain, we first filtered 9320 candidate structures from the Materials Project [28] (MP). Representative samples were then selected via coverage‐oriented farthest‐point sampling, yielding 1735 structures for explicit MCA labeling. We further augmented the dataset with 1027 uniaxial entries from Novamag [29], resulting in a final set of 2762 labeled structures. Full selection criteria, embedding procedures, and deduplication details are provided in Section S3.

While K_eff_ = (E_⊥_ − E_∥_)/V for uniaxial systems recovers K_1_ exactly at second order (E_⊥_ and E_∥_ denote the spin–orbit‐coupled energies for magnetization perpendicular and parallel to the easy axis, respectively), the three‐axis protocol K_eff_ = (E_2_ − E_1_)/V for low‐symmetry systems is an approximation whose physical justification and quantitative accuracy require examination. Specifically, the identification of K_eff_ with the minimum magnetization‐reversal barrier relies on symmetry arguments that become progressively less constraining from orthorhombic (exact under second‐order anisotropy) to monoclinic (exact for b‐axis; ac‐plane requires angular sampling) to triclinic (no symmetry constraints). We validate this approximation through dedicated DFT calculations across 35 representative structures spanning all three low‐symmetry classes; the results are presented in Section 3.1.

### 2.2 Training of MCA Prediction Models (Classification and Regression) and Generative Model

Our predictive modeling followed a two‐stage workflow motivated by the symmetry‐dependent definition of K_eff_. A binary classifier was first trained on uniaxial systems to determine the sign of the anisotropy (easy‐axis vs. easy‐plane). In these systems, K_eff_ = (E_a_ − E_c_)/V can be negative, corresponding to an easy‐plane preference, making classification meaningful. In contrast, for triclinic, monoclinic, and orthorhombic structures, the three‐axis descriptor K_eff_ = (E_2_ − E_1_)/V is non‐negative by construction and quantifies the energy separation between the two lowest sampled crystallographic‐axis orientations. Therefore, classification was applied only to the uniaxial subset. This restriction is also physically motivated: for low‐symmetry crystal systems, no symmetry‐enforced easy‐plane state exists—the anisotropy energy surface always retains a preferred direction, with the plane perpendicular to the easy axis remaining nonisotropic.

Second, a regressor was trained to predict the magnitude of easy‐axis MCA. The regression set was therefore composed of structures expected to have non‐negative anisotropy: triclinic, monoclinic, and orthorhombic systems, uniaxial systems classified as having positive MCA, and cubic systems. The regression targets were log‐transformed to handle values spanning several orders of magnitude, with a small offset applied to include the K_eff_ = 0 labels from cubic systems. Performance was estimated via 5‐fold cross‐validation, reporting metrics on concatenated out‐of‐fold predictions. Of the 2762 labeled structures, 1323 belong to uniaxial crystal systems and form the classification training set (*n* = 1323). The regression set (*n* = 2077) comprises the uniaxial easy‐axis subset, all non‐uniaxial low‐symmetry structures, and 914 cubic structures assigned K_eff_ = 0; uniaxial easy‐plane structures are excluded from regression as they are not target candidates. This sequential workflow of applying the classifier followed by the regressor is schematically illustrated in Figure 1.

To capture the chemical and structural diversity of the candidate space, we constructed a comprehensive descriptor set spanning stoichiometric, compositional, and structural information, including both handcrafted features and learned crystal graph convolutional neural network (CGCNN) [30] embeddings. These descriptors provide a unified representation suitable for both classification and regression tasks using random‐forest models. A complete list of features and construction details is provided in Section S4. Grouped feature importance analysis (Section S11 and Tables S8 and S9) shows that CGCNN graph embeddings contribute 16.9% of total importance despite comprising only 32 of 632 features (2.8× higher per‐feature importance than compositional features), demonstrating the complementary role of graph‐based local environment encoding.

We fine‐tuned the MatterGen diffusion model—pretrained on ∼670 000 magnetic‐density‐labeled crystals—on the uniaxial subset of our DFT corpus, comprising 692 tetragonal, trigonal, and hexagonal structures (≤20 atoms; alex_mp_20) for which the three‐axis K_eff_ is most reliable. Joint magnetic‐density and MCA conditioning was used. Conditional generation produced 1024 candidate structures targeting high M_s_ and easy‐axis MCA; after a symmetry filter (retaining tetragonal, trigonal, and hexagonal candidates), the uniaxial candidates were relaxed with MatterSim [31] and screened for stability, uniqueness, and novelty (SUN), with stability set by a MACE [32]/SevenNet [33] energy‐above‐hull consensus, before the downstream cascade. Full details are in Section S5. We emphasize that the present work does not seek to improve predictive performance through a novel machine‐learning architecture. Rather, the methodological advance lies in introducing a physically unified descriptor that enables learning across crystal symmetries previously treated separately.

### 2.3 Unified Screening Pipeline

**FIGURE 2:**
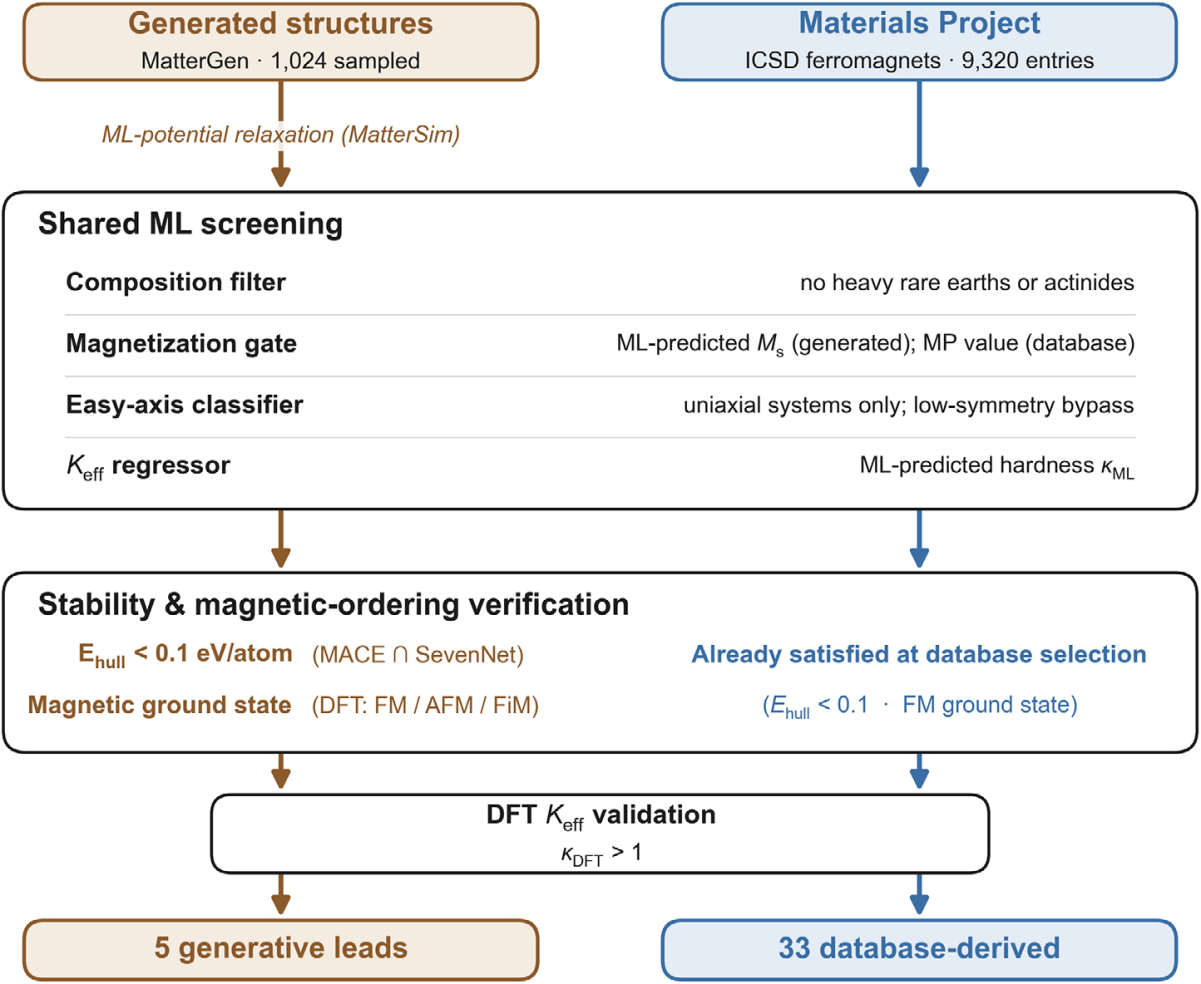
Dual‐track screening funnel. Generated (MatterGen) and MP candidates pass a shared ML screen and stability/magnetic‐ordering verification before DFT K_eff_ validation, yielding 5 generative and 33 database‐derived rare‐earth‐free or ‐lean hard magnet candidates.

A single ML pipeline screens both MP entries and generated structures (Figure 2). Generated structures are first relaxed with the MatterSim interatomic potential; both streams then pass a shared screen—compositional filtering (excluding heavy rare‐earth (Eu–Lu) and actinide elements), a saturation‐magnetization gate, an easy‐axis classifier (uniaxial structures only; low‐symmetry structures bypass it), and a K_eff_ regressor that assigns the ML hardness κ_ML_. Because generated structures lack DFT stability and ordering data, they additionally pass a MACE/SevenNet energy‐above‐hull consensus and explicit collinear magnetic‐ordering enumeration [34] (ferromagnetic, antiferromagnetic, and per‐species ferrimagnetic orderings via pymatgen, seeded from CHGNet [35] site moments, whose predicted magnetization is an upper bound on M_s_). Both streams converge at DFT validation (κ_DFT_ > 1) with magnetic‐ground‐state confirmation and phonon stability for the leads. Screening thresholds and buffer values are given in Section S14.

### 2.4 Statistical Analysis

All predictive models were random‐forest classifiers and regressors implemented in scikit‐learn. Performance was estimated by 5‐fold cross‐validation (stratified for classification), with metrics computed on concatenated out‐of‐fold predictions and reported as mean ± standard deviation across folds (classification, *n* = 1323; regression, *n* = 2077; no data excluded). Model quality is quantified by predictive metrics (precision, recall, F1, R^2^).

## 3 Results

### 3.1 Physical Validation of K_eff_ for Low‐Symmetry Crystal Systems

The effective anisotropy constant K_eff_ = (E_2_ − E_1_)/V defined for low‐symmetry crystal systems (orthorhombic, monoclinic, triclinic) requires physical justification, since the easy‐axis direction is not constrained to coincide with the a, b, or c crystallographic axes in general. In this section, we provide symmetry‐based proofs for each crystal class and present DFT validation results. The 35 validation structures were drawn directly from the labeled subset of the main screening dataset, spanning K_eff_ = 0.10–38.6 MJ/m^3^ across orthorhombic, monoclinic, and triclinic structures to ensure unbiased coverage of the full dynamic range rather than selective sampling of favorable cases.

**FIGURE 3:**
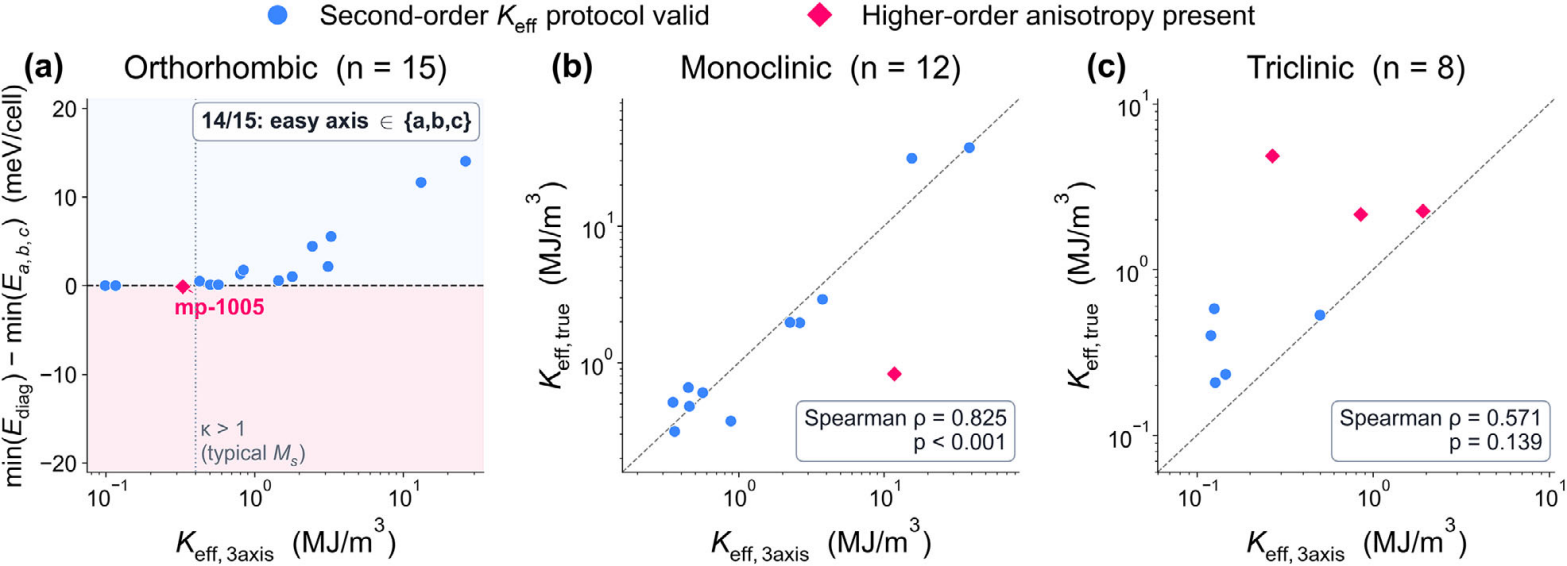
Validation of the three‐axis K_eff_ protocol for low‐symmetry crystal systems. (a) Orthorhombic (*n* = 15): minimum diagonal‐direction energy minus minimum crystallographic‐axis energy; ΔE > 0 confirms the easy axis lies within {a, b, c} (14/15; the exception mp‐1005 reflects dominant fourth‐order anisotropy at very small K_eff_). (b) Monoclinic (*n* = 12) and (c) triclinic (*n* = 8): K_eff, 3axis_ vs. K_eff, true_; dashed lines denote y = x.

For orthorhombic systems (222, mm2, mmm point groups), group‐theoretical analysis shows that the symmetry operations of each point group — two‐fold rotation axes in 222, mirror planes and a C_2_ axis in mm2, and three mutually perpendicular mirror planes in mmm — independently force all off‐diagonal second‐order anisotropy tensor elements to zero (K_ab_ = K_ac_ = K_bc_ = 0). The anisotropy energy is therefore diagonal in the crystallographic frame, E/V = K_aa_ s_a_^2^ + K_bb_ s_b_^2^ + K_cc_ s_c_^2^ (where s_a_, s_b_, and s_c_ denote the direction cosines of the magnetization along a, b, and c, respectively), so that a, b, and c coincide with the eigenvectors of the K tensor and the energy extrema on the unit sphere are confined to {a, b, c} (see Section S2.1 for the point‐group proofs and the saddle‐point derivation of K_eff_ = (E_b_ − E_a_)/V). Assuming E_a_ < E_b_ < E_c_, the minimum coherent‐rotation path from +a to −a traverses the equatorial plane at the saddle‐point energy E_b_—not the hard axis E_c_—yielding K_eff_ = (E_b_−E_a_)/V as the minimum reversal barrier within this second‐order description. DFT validation across 15 orthorhombic structures confirms this: SOC energies were computed along both the three crystallographic axes {a, b, c} and four additional diagonal directions ([110], [101], [011], [111]); in 14 of 15 structures, the global energy minimum lies along a crystallographic axis rather than any diagonal direction (Figure 3a). The single exception (mp‐1005, K_eff_ = 0.33 MJ/m^3^) exhibits fourth‐order anisotropy comparable to the second‐order term. Crucially, all structures above the practical screening threshold (K_eff_ > 0.4 MJ/m^3^, the κ = 1 boundary for typical magnetization ≈ 0.7 T) conform to the second‐order prediction, so this exception has no consequence for screening outcomes.

For monoclinic systems (2, m, 2/m point groups), the symmetry operations of each point group—a two‐fold rotation axis along b (point group 2), a mirror plane perpendicular to b (point group m), or both (point group 2/m)—independently force K_ab_ = K_bc_ = 0 while leaving K_ac_ unconstrained. As a result, b is always an eigenvector of the K tensor (with eigenvalue K_bb_), while the remaining two eigenvectors lie within the ac‐plane at an angle determined by K_aa_, K_cc_, and K_ac_. The easy axis is therefore either the b‐axis or an ac‐plane direction, depending on which eigenvalue is smallest (see Section S2.2 for the full derivation). The ac‐plane energy follows E(θ)/V = E_0_ + A·sin^2^(θ − θ_min_), enabling extraction of K_eff, true_ from 8‐point DFT sampling (θ = 20°, 40°, …, 160°) in the ac‐plane. Here, E_0_ is the baseline energy density at the easy‐axis minimum and A sets the anisotropy amplitude. The Spearman rank correlation coefficient ρ quantifies the degree to which K_eff, 3axis_ preserves the rank ordering of K_eff, true_ (p is the probability of observing ρ ≥ this value by chance under the null hypothesis of no correlation). Across 12 monoclinic structures, the three‐axis protocol K_eff, 3axis_ achieves a Spearman rank correlation of *ρ* = 0.825 (*p* = 0.001, *n* = 12) against K_eff, true_, demonstrating rank preservation sufficient for high‐throughput screening (Figure 3b). One structure (mp‐1104676) exhibits a double‐minimum energy landscape in the ac‐plane (Figure S2), indicating that fourth‐order anisotropy terms are non‐negligible—a condition under which the sin^2^(θ − θ_min_) approximation breaks down. Notably, such deviations from the single‐harmonic model are rare (1 of 12 structures), and even including this outlier, the rank correlation remains *ρ* = 0.825, suggesting that the three‐axis protocol retains its utility as a trend indicator even when the functional form is not strictly satisfied.

For triclinic systems (P1, P¯1), which carry no symmetry constraints on the K tensor, we employ a 6‐parameter second‐order anisotropy tensor fit to 19‐direction DFT sampling (16 Fibonacci hemisphere directions + 3 crystallographic axes). Eigenvalue decomposition of the fitted tensor yields K_eff, true_ = (λ_2_ − λ_1_)/V, where λ_1_ ≤ λ_2_ ≤ λ_3_ are the eigenvalues sorted in ascending order (see Section S2.3 for the detailed derivation). Across 8 triclinic structures, the three‐axis protocol yields *ρ* = 0.571 (*p* = 0.139, *n* = 8), indicating only moderate rank preservation (Figure 3c). Given that triclinic structures constitute just 6.6% of the unlabeled pool and 1.4% of the labeled set, this limitation has a restricted impact on the overall screening workflow. Full‐sphere sampling with tensor fitting (as used in this validation) is recommended for confirmed high‐priority triclinic candidates at the DFT validation stage.

Taken together, these results establish K_eff_ as a physically grounded screening descriptor across all crystal classes: exact within the second‐order anisotropy approximation for orthorhombic systems, rank‐preserving for monoclinic systems (*ρ* = 0.825, *p* = 0.001), and sufficiently informative for coarse‐grained screening, albeit not yet for quantitative prediction for triclinic systems. Importantly, this validation is what enables the low‐symmetry hard‐magnet discoveries reported in Section 3.3: without a descriptor identically defined across all seven crystal systems, orthorhombic and monoclinic candidates such as those identified in this study could not have been entered into a single ML screening pipeline, and would have remained outside the scope of most prior family‐ or uniaxial‐restricted approaches.

### 3.2 Performance of the MCA Prediction Models

#### 3.2.1 Classification model (Screening for Easy‐Axis Candidates)

To minimize unnecessary DFT costs, we utilize the classifier to filter out clear easy‐plane candidates. Given that missing a promising easy‐axis material is far more detrimental than admitting false positives, we adopted a deliberately high‐recall screening strategy. To determine an optimal decision threshold, we analyzed the precision–recall curve from 5‐fold out‐of‐fold predictions (Figure S5). An F1‐optimal operating point yielded a threshold of τ = 0.302 (Figure S5), designed to filter out only the most confidently predicted easy‐plane systems. A full threshold sensitivity analysis across τ = 0.1–0.7, including false‐positive and miss‐rate breakdowns, is provided in Section S10 (Table S7).

#### 3.2.2 Regression Model (Easy‐Axis MCA Magnitude)

**FIGURE 4:**
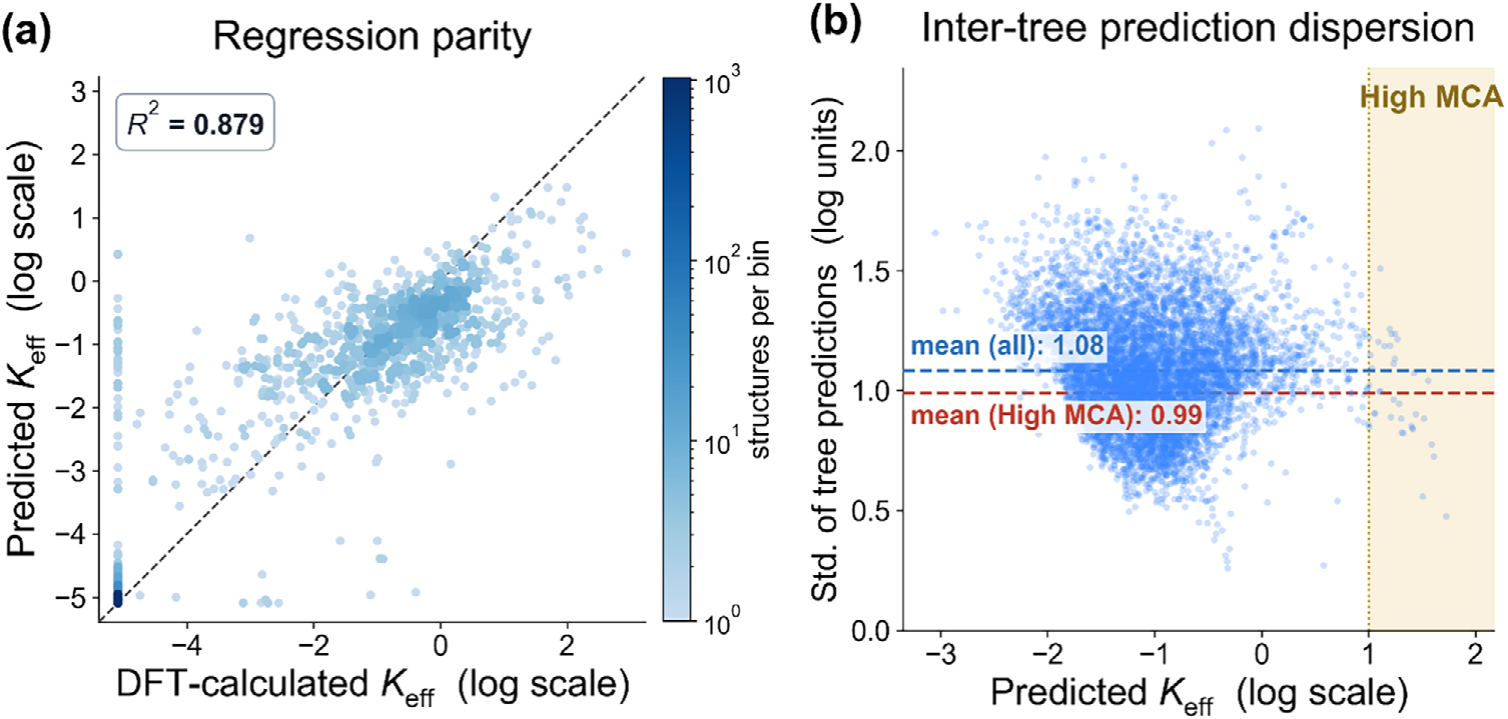
(a) Parity plot for the regression model predicting log(K_eff_). Points are colored by local density (structures per bin). (b) Scatter plot of predicted K_eff_ vs. inter‐tree prediction dispersion, showing that structures predicted to have high K_eff_ tend to exhibit lower inter‐tree dispersion; dashed lines mark the mean dispersion of all structures (blue) and of the high‐MCA region (log K_eff_ > 1, shaded; red).

Predicting K_eff_ across diverse chemistries and symmetries is challenging, particularly given the approximate nature of the labels produced by our three‐crystallographic axis protocol. Nonetheless, the Random Forest regressor effectively captures the dominant trends, achieving an R^2^ = 0.8779 ± 0.0196 on 5‐fold out‐of‐fold predictions (Figure 4a). The parity plot shows that predictions closely follow the diagonal, with only minor deviations, including a slight overestimation of near‐zero cubic entries—an inconsequential effect since these systems are prescreened by symmetry.

As a model‐internal consistency check, we examined the inter‐tree prediction dispersion, computed as the standard deviation across the forest's decision trees (Figure 4b). In the high‐K_eff_ region, which is critical for identifying hard magnets, this dispersion is lower on average than in the overall population. Because inter‐tree dispersion is not a calibrated estimate of predictive uncertainty—particularly for candidates lying far from the training distribution—we use it only as a model‐internal stability indicator and rely on explicit DFT calculations for candidate validation.

### 3.3 Screening Outcomes and Significance

**FIGURE 5:**
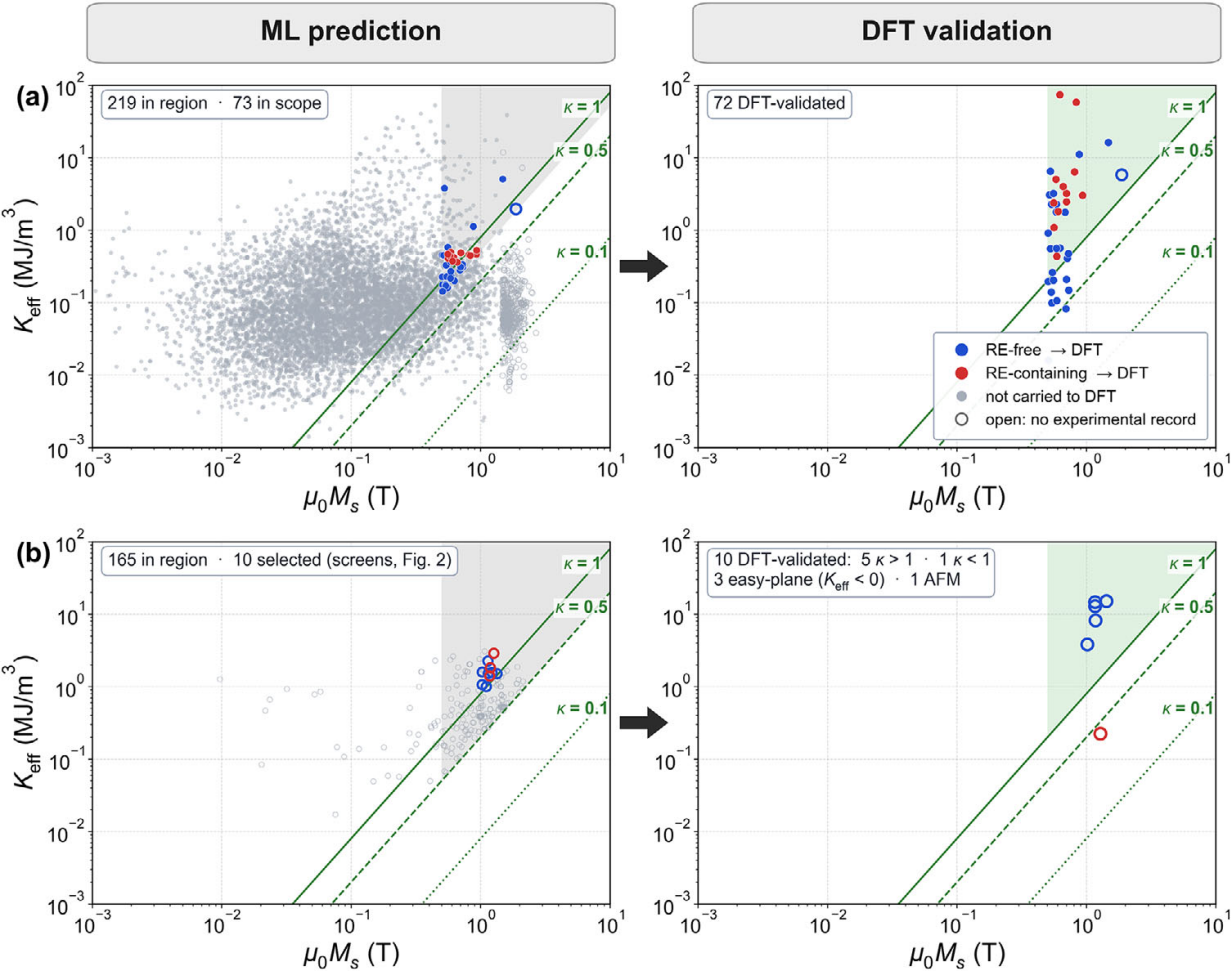
Screening on the K_eff_–µ_0_M_s_ plane using the hardness parameter κ, before (left) and after (right) DFT validation; green shading marks κ > 1. Shaded gates in the left panels: µ_0_M_s_ > 0.5 T with κ > 0.8 (a, MP pool) and κ > 0.5 (b, generated pool). Colored points were carried to DFT; grey points were screened out by the composition scope in (a) and by the stability and ordering screens of Figure 2 in (b). Open markers denote entries without experimental record; the sole such candidate carried to DFT (Fe_3_Pt) appears as the open blue circle.

Of the 1735 DFT‐labeled structures used to train the regressor, 12 already satisfy the rare‐earth‐free or ‐lean composition, magnetization, and κ > 1 criteria (Figure S6). Applying the two‐stage classifier–regressor to the 9320 experimentally reported MP ferromagnets passed 73 to DFT validation, of which 21 exceed κ = 1 (Figure 5a). Together, these give 33 rare‐earth‐free or ‐lean hard magnet candidates (12 from the labeled set, 21 newly identified by the screen; Tables S3 and S4). These 33 recover the textbook L1_0_ permanent magnets FePt [36], CoPt [37], MnAl [38], MnGa [39, 40] and FeNiPt_2_ [41], together with the double‐perovskite ferrimagnet Sr_2_FeMoO_6_ [42], confirming that the unified screening workflow reproduces accepted benchmarks rather than drifting toward artefacts.

**FIGURE 6:**
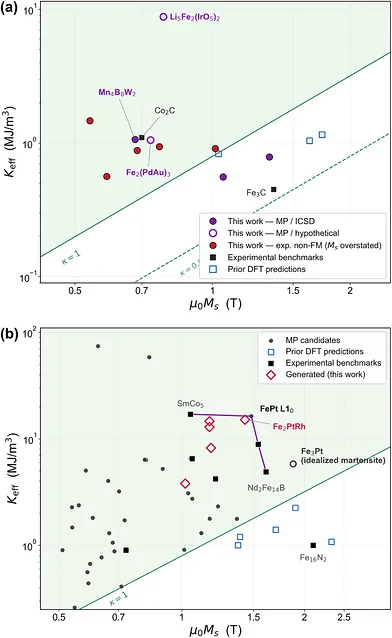
Magnetization–anisotropy landscape of the candidate hard magnets. (a) Low‐symmetry (orthorhombic and monoclinic) candidates: purple, this work (filled, MP/ICSD; open, hypothetical); red, candidates whose MP ferromagnetic label is contradicted by experiment (µ_0_M_s_ overstated); open blue squares, prior DFT predictions (Halder, 2025; Chelikowsky, 2024); black squares, experimental benchmarks (Coey, 2016). Green lines mark κ = 1 and κ = 0.5; shading, the hard‐magnet regime. (b) Global landscape: the 33 MP candidates (dark circles; the five literature‐contradicted entries appear in (a)), the five generated rare‐earth‐free hard‐magnet candidates (crimson diamonds), and the idealized‐martensite Fe_3_Pt (open circle). The purple line traces the Pareto front of the benchmark and validated magnets. Benchmark values are experimental and serve as qualitative references for the DFT‐predicted candidates.

The descriptor is most impactful in the low‐symmetry regime, where the uniaxial‐anisotropy assumption breaks down—a regime that prior searches have not typically addressed. High magnetization is not the scarce resource in this regime—the MP database holds thousands of low‐symmetry entries with computed µ_0_M_s_ above 0.7 T—whereas anisotropy strong enough to hold that magnetization (κ > 1) is rare; it is this bottleneck that the screen targets. By avoiding fixed composition‐family and uniaxial‐symmetry pre‐filters, the screen attains κ > 1 across orthorhombic and monoclinic borides, ferrites, selenides, tellurides and oxides (Figure 6a)—a region the prior non‐uniaxial literature, bounded by carbide and Fe–Co–X chemistries at or below κ = 1 [1, 13], has barely entered [17, 22, 23, 24]. Six of the 33 candidates lie here, and because their magnetic order is inherited from collinear‐ferromagnetic database labels—computed descriptors, not ground states [34]—we check each against the literature, a necessary safeguard when screening on database magnetic labels. Co_6_B_4_O_12_ [43, 44], Sr_6_Fe_4_O_10_ [45] and Na_6_Mn_8_Te_12_ [46] are antiferromagnetic and Cr_2_Fe_4_Se_8_ [47] and Ca_2_FeOsO_6_ [48] ferrimagnetic (Figure 6a, red), leaving Mn_4_B_8_W_2_ as the only low‐symmetry MP candidate whose magnetic ordering is not contradicted by reported experimental or computational evidence. A broadened search over hypothetical structures further surfaces a metallic and an oxide candidate—Fe_2_(PdAu)_3_ (κ = 1.57), a robust low‐symmetry hard ferromagnet, and the higher‐anisotropy Li_5_Fe_2_(IrO_5_)_2_, whose ferromagnetic label is likewise only a computed descriptor (Figure 6a). For triclinic structures, the three‐axis protocol functions as a coarse screening filter rather than a quantitative ranking (*ρ* = 0.571; Section 3.1), and triclinic candidates surfacing in future screens should be validated using the full‐sphere tensor fit of Section 3.1 in place of the three‐axis protocol. The present shortlists are unaffected: none of the candidates in Tables S3 and S4 is triclinic.

Conditioning the generative model on anisotropy shifts the ML‐predicted K_eff_ distribution of the sampled structures upward relative to magnetization‐only conditioning (Figure S4)—a population‐level trend that the validation pipeline below converts into DFT‐confirmed candidates. A staged cascade—ML easy‐axis and hardness screening, a MACE/SevenNet stability consensus, explicit magnetic‐ordering enumeration, and DFT validation closed by full spin–orbit anisotropy—reduced the 1024 generated candidates to five DFT‐confirmed rare‐earth‐free candidates satisfying the intrinsic κ > 1 screening criterion (Figure 5b): Fe_2_PtRh, MnCoPt_2_, two distinct FeCoPt_2_ orderings, and TaFe_3_ (per‐stage counts in Section S14). Performing the ordering enumeration before DFT is essential: it removes structures whose collinear‐ferromagnetic moment collapses under a lower‐energy antiferromagnetic ground state—the same failure mode guarded against in the MP screen. All five are metastable ordered intermetallics (formation energies 35–160 meV/atom above the convex hull, a positioning shared by L1_0_ FePt itself). Phonon calculations on the three lead candidates—Fe_2_PtRh, the I4/mmm FeCoPt_2_ ordering, and TaFe_3_—confirm dynamical stability with no imaginary modes, so they are presented as dynamically stable metastable candidates that may be accessible under non‐equilibrium synthesis conditions.

Among the generated candidates, Fe_2_PtRh stands out, with K_eff_ = 15.1 MJ/m^3^ at µ_0_M_s_ = 1.43 T—anisotropy approaching that of FePt L1_0_ at comparable magnetization, entirely rare‐earth‐free. On the global M_s_–K_eff_ landscape (Figure 6b) the generated candidates sit just inside the benchmark Pareto front defined by SmCo_5_, FePt L1_0_, Sm_2_Fe_17_N_3_ and Nd_2_Fe_14_B, while exceeding the prior rare‐earth‐free computational frontier [17, 24], which tops out near 2.25 MJ/m^3^, by roughly sevenfold. Beyond this front lies Fe_3_Pt (open circle), a known Fe–Pt martensite whose idealized Bain‐distorted, fully‐ordered structure computes to κ_DFT_ = 1.44 at µ_0_M_s_ ≈ 1.9 T. Because electron diffraction shows the real martensite carries additional atomic displacements beyond this high‐symmetry idealization [49, 50], the realized anisotropy may differ from this idealized value, and we present Fe_3_Pt as an idealized‐structure reference for the high‐magnetization frontier rather than a synthesis target. The natural application is heat‐assisted magnetic recording, where FePt L1_0_ is the incumbent medium, Pt content is standard practice, and the governing figure of merit is the thermal‐stability factor K_eff_V/k_B_T rather than the bulk energy product [36]; there, Fe_2_PtRh is a credible rare‐earth‐free alternative approaching the anisotropy of the incumbent.

In terms of novelty, we found no prior reports of TaFe_3_ (free of both rare‐earth and noble metals), the Fe_2_PtRh polytype, or the I4/mmm FeCoPt_2_ ordering as ordered ferromagnetic phases, whereas the second FeCoPt_2_ ordering corresponds to a known structure (mp‐1224993) that the generator recovered independently. A compositional‐distance analysis based on element overlap and stoichiometric similarity shows that the four Pt‐bearing candidates lie closest to the established L1_0_ platinum magnets FePt and CoPt, indicating local refinement of a known hard‐magnet chemistry. TaFe_3_, by contrast, is a clear outlier: its nearest MP hard magnet shares only Fe, and no MP hard magnet contains Ta. To relate the generated candidates to the training data directly, Section S13 additionally reports, for each candidate, the nearest compositional and structural neighbors in the 692‐structure MatterGen fine‐tuning set and the full 2762‐structure MCA‐labeled dataset, evaluated with structural‐fingerprint similarity in addition to compositional distance. None of the five candidates matches any training‐set structure under pymatgen's StructureMatcher, and the four Pt‐bearing candidates occur in neither training set at their exact compositions. Their nearest structural neighbors across all three reference sets are L1_0_‐type orderings and their layered derivatives (FePt, FeNiPt_2_, and the isostructural prototypes NiPt, TiHg, and TiCdHg_2_; Table S11). TaFe_3_ is a structural outlier: its fingerprint distance to the nearest training structure lies at the 90th percentile of the training set's own nearest‐neighbor distances, and the only TaFe_3_ polymorph present in the fine‐tuning data—a tetragonal easy‐plane phase—is structurally unrelated to the generated easy‐axis P3m1 candidate (Table S11 and Figure S11). The generated candidates therefore represent two complementary discovery modes: local refinement of established hard‐magnet chemistries and in TaFe_3_, a candidate compositionally distinct from all MP‐derived hard magnets and structurally novel relative to the training data (Section S13). Taken together, the database‐screening and generative routes provide a two‐part demonstration of the framework: methodological breadth, by reaching κ > 1 candidates across orthorhombic and monoclinic classes that most previous uniaxial‐restricted screening studies did not address; and candidate generation, by identifying rare‐earth‐free leads such as Fe_2_PtRh and TaFe_3_. Fe_2_PtRh delivers benchmark‐class intrinsic anisotropy and magnetization approaching FePt L1_0_ while remaining rare‐earth‐free, whereas TaFe_3_ provides a fully noble‐metal‐free lead with no close MP analogue; both are phonon‐stable.

## 4 Conclusions

In this study, we introduced a unified effective anisotropy constant K_eff_ applicable across all seven crystal systems, substantially extending hard‐magnet screening beyond conventional symmetry‐restricted approaches. Combined with composition‐independent screening of the MP and a fine‐tuned diffusion‐based generative model, this framework identifies 33 κ > 1 hard magnet candidates from the MP screen, 28 of which survive a literature check of their reported magnetic ordering. It further proposes five rare‐earth‐free generated candidates, the three lead structures among them confirmed dynamically stable by phonon calculations.

Crucially, K_eff_ is constructed to approximate the minimum energy barrier for coherent magnetization reversal—the saddle‐point energy relative to the easy‐axis minimum—which is identical to K_1_ in uniaxial systems at the second‐order level and provides a symmetry‐dependent approximation for low‐symmetry systems, as quantified in Section 3.1. This formulation allows κ = √(K_eff_/µ_0_M_s_^2^) to be used as a common screening parameter across crystal symmetries, enabling a symmetry‐spanning hard‐magnet discovery workflow.

The present work contributes primarily in two respects. First, extension of physically grounded screening across crystal symmetries: the unified K_eff_ reaches κ > 1 across orthorhombic and monoclinic classes that family‐ and uniaxial‐restricted ML pipelines generally exclude, charting a non‐uniaxial region that standard surveys leave essentially unexplored. Second, generation of experimentally testable rare‐earth‐free candidates: among the generative proposals, Fe_2_PtRh reaches benchmark‐class anisotropy (K_eff_ = 15.1 MJ/m^3^) and magnetization (µ_0_M_s_ = 1.43 T)—approaching the incumbent FePt L1_0_—without any rare‐earth element, and is confirmed dynamically stable by phonon calculation. The five generated candidates divide into two kinds: the four Pt‐bearing candidates are orderings and polytypes within an established hard‐magnet chemistry, whereas TaFe_3_ reaches a composition family free of both rare‐earth and noble metals—a trigonal P3m1 polymorph whose easy‐axis character is absent from the only TaFe_3_ structure seen during fine‐tuning (Section S13 and Figure S11). The successful rediscovery of established L1_0_ hard magnets (FePt, CoPt, MnAl, MnGa, FeNiPt_2_) and of the ferrimagnet Sr_2_FeMoO_6_ confirms that the screen recovers known benchmarks, lending credibility to the newly identified low‐symmetry and generative candidates as experimental follow‐up targets.

Several limitations indicate directions for future work. (i) The three‐axis K_eff_ protocol is formally exact within the second‐order anisotropy approximation for orthorhombic systems and rank‐preserving for monoclinic systems. For triclinic structures, however, the crystallographic axes need not coincide with the principal axes of the anisotropy tensor, so three‐axis energies only partially constrain the reversal barrier. Higher‐order anisotropy or denser angular sampling will likely be necessary for quantitatively accurate anisotropy estimates in triclinic systems. Accordingly, we view the present descriptor primarily as a practical screening metric rather than a rigorous anisotropy constant for this crystal class. (ii) Curie temperature was not included; composition‐based T_c_ predictors [51, 52] can be integrated for a more comprehensive screen. (iii) The current MatterGen implementation is restricted to primitive cells with ≤20 atoms, excluding complex permanent magnets such as Nd_2_Fe_14_B; extending generative approaches to larger unit cells is a key direction. (iv) MatterSim provided fast geometry relaxation of generated structures ahead of DFT; higher‐fidelity universal ML interatomic potentials would further improve the accuracy of this pre‐relaxation. (v) Expanding the fine‐tuning corpus beyond the present 692 structures and extending phonon validation across the full candidate set are natural routes to broaden generative coverage.

We therefore view the present framework not as a replacement for existing hard‐magnet discovery strategies, but as a complementary platform that broadens the accessible search space across crystal symmetry and composition. Future advances in machine‐learning architectures can be incorporated directly into this framework without modifying the underlying anisotropy descriptor.

## Conflicts of Interest

The authors declare no conflicts of interest.

## Supporting information




**Supporting File**: advs77553‐sup‐0001‐SuppMat.docx.

## Data Availability

The machine‐learning pipeline, trained models, and analysis code are publicly available at https://github.com/kimhj520/unified‐anisotropy‐hard‐magnets under the PolyForm Noncommercial License 1.0.0. The five DFT‐confirmed generated candidate structures, processed structure–property dataset, and fixed cross‐validation splits sufficient to retrain and evaluate the reported models are available under the Creative Commons Attribution‐NonCommercial 4.0 International (CC BY‐NC 4.0) license. The raw DFT calculation files cannot be redistributed under the industrial collaboration agreement but are available from the corresponding author upon reasonable request. Crystal structures were obtained from the Materials Project, and the uniaxial‐anisotropy reference data from the Novamag database.
