# Supplementary material for: Broadening Hard‐Magnet Discovery Beyond Symmetry Constraints via Unified Effective Anisotropy
Source: Adv Sci (Weinh). 2026 Sep 8:e24393. Online ahead of print. doi: 10.1002/advs.202524393 (PMC13554380; doi:10.1002/advs.202524393)
Supplement: Supplementary file 1 [file ADVS-9999-e24393-s001.docx]

**Supporting Information**

**Broadening Hard-Magnet Discovery Beyond Symmetry Constraints via Unified Effective Anisotropy**

Hojae Kim^a^, Hyeondeok Shin^d^, Kyungju Nam^c^, Seunghyo Noh^c^, Donghwi Kim^c^ and Yousung Jung^*,a,b^

^a^ Department of Chemical and Biological Engineering, and Institute of Chemical Processes, Seoul National University, 1 Gwanak-ro, Gwanak-gu, Seoul 08826, Republic of Korea

^b^ Institute of Engineering Research, Seoul National University, 1 Gwanak-ro, Gwanak-gu, Seoul 08826, Republic of Korea

^c^ Materials Research & Engineering Center, AVP Division, Hyundai Motor Company

^d^ Computational Science Division, Argonne National Laboratory, Lemont, Illinois 60439, United States

**E-mail address:* [yousung.jung@snu.ac.kr](mailto:kimhojae@snu.ac.kr)

**1. Details of Magnetocrystalline anisotropy (MCA) Calculation and Element-Specific Settings**

We computed $E_{MCA}$ using Density Functional Theory (DFT) with **spin–orbit** coupling (SOC) and the projector augmented-wave (PAW) method^1,2^ as implemented in Vienna *Ab initio* Simulation Package (VASP). Within Kohn-Sham scheme, the Perdew-Burke-Ernzerhof (PBE) parameterization^3^ was employed for the exchange-correlation functional. Strongly correlated *f*-electrons were treated with an on-site Hubbard U correction within the DFT framework^4^. For the lanthanide series (La–Lu), we adopted the Hubbard-I based parameterization proposed by Locht *et al.* with a uniform U=7 eV and element-dependent Hund exchange J values^5^. For early actinides (Ac–Pu), U and J were selected from element-specific literature^6–9^ to reproduce known electronic/magnetic properties. For the transition metal elements, semi-core *p* states were included in the PAW pseudopotentials to account for strong core-valence correlations.
 To balance accuracy and throughput, compounds containing lanthanides or early actinides were treated with a plane-wave cutoff of **700 eV** and **Γ-centered** k-point meshes with a density of **20,000 k-points per reciprocal atom**. For all other structures we used **520 eV** and a target density of **2,000 k-points**. Γ-centered meshes were generated automatically using **pymatgen^1^**^0^. All magnetocrystalline anisotropy (MCA) values were obtained from **single-point** total energy differences on relaxed geometries from **Materials Project (MP) database**. We first converged the charge density using **collinear spin-polarized calculation** to obtain a reference electronic ground state. Using that fixed density, we then evaluated total energies including SOC and the spin-quantization axis aligned sequentially along the **conventional crystallographic axes a, b, and c**, resulting three orientations per structure.

# **2. Physical Validation of K_eff_ for Low-Symmetry Systems**

The following sections present detailed symmetry-based derivations and DFT validation of the K_eff_ descriptor for orthorhombic (Section 2.1), monoclinic (Section 2.2), and triclinic (Section 2.3) crystal systems. A summary of the key validation results is provided in Section 3.1 of the main text.

## **2.1. Orthorhombic Systems (222, mm2, mmm point groups)**

The second-order MCA energy in an arbitrary reference frame can be written as:

E(ś)/V = Σ_ij_ K_ij_ s_i_ s_j_	(S1)

where ŝ = (s_a_, s_b_, s_c_) is the unit spin vector and K_ij_ is the anisotropy tensor^11^. The orthorhombic crystal system comprises three point groups (222, mm2, mmm), all of which force the anisotropy tensor to be diagonal in the crystallographic frame. We demonstrate this for the highest-symmetry representative, mmm, which possesses three mutually perpendicular mirror planes.

Written in matrix form, K is the real symmetric 3×3 anisotropy tensor relating the spin-orbit energy to the spin direction:

$K = \left( \begin{matrix} K_{\mathrm{aa}} & K_{\mathrm{ab}} & K_{\mathrm{ac}} \\ K_{\mathrm{ab}} & K_{bb} & K_{bc} \\ K_{\mathrm{ac}} & K_{bc} & K_{\mathrm{cc}} \end{matrix} \right)$	(S2)

where Kᵢⱼ = Kⱼᵢ by time-reversal symmetry of the spin-orbit energy.

The mirror operation $M_{a}$ acts on the spin vector as $s \to M_{a}s$, where $M_{a} = diag(-1, 1, 1)$. Invariance of $E$ under this operation requires $K = M_{a}^{T}KM_{a}$. Computing the product explicitly gives:

$M_{a}^{T}KM_{a} = \left( \begin{matrix} K_{\mathrm{ss}} & -K_{\mathrm{ab}} & -K_{\mathrm{ac}} \\ -K_{\mathrm{ab}} & K_{\mathrm{bb}} & K_{\mathrm{bc}} \\ -K_{\mathrm{ac}} & K_{\mathrm{bc}} & K_{\mathrm{cc}} \end{matrix} \right)$	(S3)

Equating to K entry-by-entry forces $K_{\mathrm{ab}} = 0$ and $K_{\mathrm{ac}} = 0$. Applying $M_{b} = diag(1, -1, 1)$ analogously yields $K_{\mathrm{bc}} = 0$, leaving the tensor fully diagonal in the crystallographic frame.

The physical significance — that the easy axis is confined to {a, b, c} — follows from a Lagrange-multiplier argument. The anisotropy energy E(ŝ) = ŝᵀ Kŝ is extremized on the unit sphere |ŝ| = 1. The stationarity condition is:

$\nabla_{\hat{s}}\left[ \hat{s}^{\top} K \hat{s} - \lambda\left( \left| \hat{s} \right|^{2} - 1 \right) \right] = 0$	(S4)

Computing the gradient with respect to ŝ:

$2K \hat{s} - 2\lambda\hat{s} = 0 \Longrightarrow K \hat{s} = \lambda\hat{s}$	(S5)

Equation (S5) is the eigenvalue equation for K: every extremal direction ŝ* on the unit sphere is an eigenvector of K, with λ equal to the corresponding energy density E(ŝ*)/V. Because the off-diagonal elements all vanish for orthorhombic symmetry (established above), K = diag(K_aa_, K_bb_, K_cc_) is diagonal in the crystallographic frame. The eigenvectors of a diagonal matrix are exactly the standard basis vectors — here the crystallographic axes a, b, c. Therefore, the global energy minimum (easy axis), the saddle point, and the maximum all lie at crystallographic axes, confirming that the easy axis is constrained to {a, b, c}.

*† For the lower-symmetry orthorhombic point groups 222 and mm2, the same result follows from their two-fold rotation axes. For example, the C_2_ rotation about the a-axis acts as (s_a_, s_b_, s_c_) → (s_a_, −s_b_, −s_c_), with matrix R_a_ = diag(1, −1, −1). The invariance condition R_a_ᵀ K R_a_ = K forces K_ab_ = K_ac_ = 0; applying C_2_ about b and c similarly yields K_bc_ = 0. All three orthorhombic point groups therefore produce a fully diagonal anisotropy tensor in the crystallographic frame.*

With the tensor diagonal, the energy becomes E/V = K_aa_ s_a_² + K_bb_ s_b_² + K_cc_ s_c_². Defining θ as the polar angle measured from the easy axis (a) and φ as the azimuthal angle in the bc-plane, the spin components are s_a_ = cosθ, s_b_ = sinθ sinφ, s_c_ = sinθ cosφ. Substituting and using sin²φ = (1 − cos 2φ)/2 and cos²φ = (1 + cos 2φ)/2 gives E/V = K_aa_ + sin²θ [(K_bb_ + K_cc_)/2 – K_aa_] + sin²θ cos 2φ [(K_cc_ – K_bb_)/2]. Dropping the constant K_aa_ and identifying K_1_ = (K_bb_ + K_cc_)/2 – K_aa_ = (E_b_ + E_c_)/2 − E_a_ and K_1_' = (K_cc_ – K_bb_)/2 = (E_c_ − E_b_)/2 recovers equation (S6)^11^. These identifications also verify the two key relations: K_eff_ = K_1_ − K_1_' = E_b_ − E_a_ and K_1_ + K_1_' = E_c_ − E_a_.

The energy surface is therefore:

E(θ,φ)/V = K_1_ sin²θ + K_1_' sin²θ cos 2φ	(S6)

where K_1_ and K_1_' are the two independent second-order constants. Assuming E_a_ < E_b_ < E_c_ (easy axis = a), the magnetization-reversal path from +a to −a must traverse the equatorial plane (θ = 90°). Setting θ = 90° in equation (S6) gives sin²θ = 1, so the energy on the equatorial plane reduces directly to:

E(φ)/V = (K_1_ – K_1_') cos² φ + (K_1_ + K_1_') sin² φ	(S7)

where K_1_ − K_1_' = E_b_ − E_a_ and K_1_ + K_1_' = E_c_ − E_a_, consistent with the identifications established above. The saddle point of this equatorial profile is at φ = 0 (b-axis direction), giving:

E_saddle_ = E_b_	⇒	K_eff_ = (E_b_ − E_a_)/V = K_1_ − K_1_'	(S8)

The hardness parameter κ = √(K_eff_/μ_0_M_s_²) quantifies whether the anisotropy energy barrier resists demagnetization. For a biaxial orthorhombic system with easy axis a, the minimum-energy reversal path from +a to −a passes through the saddle point at the b-axis, yielding a barrier of E_b_ − E_a_ = K_1_ − K_1_'. Using K_1_ alone would overestimate the true barrier by K_1_' = (E_c_ − E_b_)/2. We therefore adopt K_eff_ = K_1_ − K_1_' = E_b_ − E_a_ as the physically correct and conservative estimate of the coherent-rotation barrier for orthorhombic systems, in direct analogy to K_1_ for uniaxial systems.

We note that K_eff_ here represents the coherent-rotation energy barrier in the Stoner–Wohlfarth limit, which serves as an upper bound on coercivity. The condition κ > 1 is therefore a necessary — not sufficient — criterion for hard-magnet behavior, but is the standard figure of merit for materials screening.

**DFT Validation.** To confirm that the easy axis lies within {a, b, c} for the structures in our dataset, we performed additional SOC single-point calculations along four off-axis directions ([110], [101], [011], [111]) for 15 representative orthorhombic structures spanning K_eff_ = 0.10–26.3 MJ/m³. Fig. 3(a) shows the energy differences relative to the minimum crystallographic axis energy. For 14 out of 15 structures, all off-axis energies exceed min(E_a_, E_b_, E_c_), directly confirming that the easy axis lies within {a, b, c} as required by orthorhombic symmetry at the second-order level. The single exception (mp-1005, K_eff_ = 0.33 MJ/m³) shows off-axis energies marginally below the minimum crystallographic axis energy. At this exceptionally small K_eff_, fourth-order anisotropy contributions become comparable to the second-order and can shift the easy axis off the crystallographic axes. Importantly, all structures with K_eff_ above the practical screening threshold (κ > 1 requires K_eff_ > 0.4 MJ/m³ for typical M_s_ values) conform to the second-order prediction.

**Fig. S1. Off-axis vs. crystallographic easy-axis energies for 15 orthorhombic structures (sorted by K_eff_ in descending order, left to right). Bars show DFT total energies; y-axes are independently scaled to the energy variation range of each structure. Blue: crystallographic easy axis (lowest energy); green: off-axis direction (energy above easy axis); red: off-axis direction with energy below the easy axis (exception, mp-1005).**

Figure S1 displays the DFT-calculated total energies along five magnetic directions for all 15 orthorhombic validation structures. In 14 of 15 cases all four off-axis directions ([110], [101], [011], [111]) lie above the crystallographic easy-axis energy (green bars taller than the blue bar), directly confirming the second-order symmetry argument. The single exception mp-1005 falls below the practical screening threshold and is attributed to dominant fourth-order anisotropy as discussed above.

## **2.2. Monoclinic Systems (2, m, 2/m point groups)**

The monoclinic crystal system comprises three point groups (2, m, 2/m), all of which constrain the anisotropy tensor to the same form. We demonstrate this for all three cases.

For point group 2 (C_2_ rotation about b), the rotation matrix is R_b_ = diag(−1, 1, −1), acting as (s_a_, s_b_, s_c_) → (−s_a_, s_b_, −s_c_). The invariance condition R_b_ᵀ K R_b_ = K gives:

$R_{b}^{T} K R_{b} = \left( \begin{matrix} K_{\mathrm{aa}} & -K_{\mathrm{ab}} & K_{\mathrm{ac}} \\ -K_{\mathrm{ab}} & K_{\mathrm{bb}} & -K_{\mathrm{bc}} \\ K_{\mathrm{ac}} & -K_{\mathrm{bc}} & K_{\mathrm{cc}} \end{matrix} \right)$	(S9)

Equating to K entry-by-entry forces K_ab_ = 0 and K_bc_ = 0, while K_ac_ remains unconstrained.

For point group m (mirror perpendicular to b), the reflection matrix is M_b_ = diag(1, −1, 1), acting as (s_a_, s_b_, s_c_) → (s_a_, −s_b_, s_c_). The invariance condition M_b_ᵀ K M_b_ = K gives the identical result: K_ab_ = K_bc_ = 0, K_ac_ free.

Point group 2/m contains both operations and therefore imposes the same constraints. In all three cases the anisotropy tensor takes the form:

$K = \left( \begin{matrix} K_{\mathrm{aa}} & 0 & K_{\mathrm{ac}} \\ 0 & K_{\mathrm{bb}} & 0 \\ K_{\mathrm{ac}} & 0 & K_{\mathrm{cc}} \end{matrix} \right)$	(S10)

The b-axis is always a principal axis (eigenvector) of this tensor with eigenvalue K_bb_ = E_b_. The remaining two eigenvectors lie in the ac-plane, rotated from a and c by the material-specific angle θ_min_ determined by K_ac_.

To obtain the energy profile in the ac-plane, we set s_b_ = 0 and write s_a_ = cosθ, s_c_ = sinθ, where θ is measured from the a-axis. Substituting into E/V = K_aa_ s_a_² + K_cc_ s_c_² + 2K_ac_ s_a_ s_c_ gives:

E/V = K_aa_ cos²θ + K_cc_ sin²θ + 2K_ac_ cosθ sinθ	(S11)

Converting to double-angle form using cos²θ = (1 + cos2θ)/2, sin²θ = (1 − cos2θ)/2, and sin2θ = 2cosθ sinθ:

E/V = (K_aa_ + K_cc_)/2 + (K_aa_ − K_cc_)/2 · cos2θ + K_ac_ · sin2θ	(S12)

Combining the oscillatory terms into a single cosine:

E/V = E_mid_ + R cos(2θ − 2θ_0_)	(S13)

where E_mid_ = (K_aa_ + K_cc_)/2, R = √[((K_aa_ − K_cc_)/2)² + K_ac_²], and tan(2θ_0_) = 2K_ac_/(K_aa_ − K_cc_). Note that R = (λ_+_ − λ_−_)/2, half the ac-plane eigenvalue gap.

Defining θ_min_ as the easy-axis angle (energy minimum), we have θ_0_ = θ_min_ + 90°, so cos(2θ − 2θ_0_) = −cos(2(θ − θ_min_)). Using cos(x) = 1 − 2sin²(x/2):

E/V = (E_mid_ − R) + 2R sin²(θ − θ_min_)	(S14)

Setting E_0_ = E_mid_ − R = λ_−_ and A = 2R = λ_+_ − λ_−_ recovers:

E(θ)/V = E_0_ + A · sin²(θ − θ_min_)	(S15)

where θ_min_ is the easy-axis angle in the ac-plane, and A is the anisotropy amplitude. The three relevant energy eigenvalues, sorted in ascending order, are:

[λ_−_, E_b_, λ_+_] = [E_0_, E_b_, E_0_ + A]	(S16)

or

[E_b_, λ_−_, λ_+_] = [E_b_, E_0_, E_0_ + A]	(S17)

where λ_−_ = E_0_ or E_b_ is the global energy minimum (ac-plane easy-axis), and λ_+_ = E_0_ + A is the ac-plane maximum. The true anisotropy barrier is then:

K_eff,true_ = (λ_−_ − E_b_) / V	(S18)

or

K_eff,true_ = (E_b_ - λ_−_) / V	(S19)

The three-axis protocol computes:

K_eff,3axis_ = (min(E_a_, E_c_) – E_b_) / V	(S20)

or

K_eff,3axis_ = (E_b_ - min(E_a_, E_c_)) / V	(S21)

Figure 3(b) shows that K_eff,3axis_ values cluster near the y = x line, and the Spearman rank correlation ρ = 0.825 (p = 0.001, n = 12) confirms that relative ranking is preserved, validating the screening utility of the three-axis protocol.

**DFT Validation.** We computed E(θ) at eight equally spaced angles (20°, 40°, ..., 160°) in the ac-plane for 12 monoclinic structures using noncollinear SOC calculations. The resulting E(θ) profiles were fitted to the sin² model E(θ) = E_0_ + A·sin²(θ − θ_min_) (Fig. S2) to extract θ_min_, λ_−_, and A, from which K_eff,true_ was calculated. Eleven of twelve structures show E(θ) profiles that are well described by the single-harmonic sin² model (R² > 0.80 for 11/12). The one exception, mp-1104676, exhibits two local minima in [0°, 180°], indicative of significant higher-order anisotropy beyond the second-order approximation; this structure is therefore treated as an outlier in the analysis below. The Spearman rank correlation coefficient ρ quantifies the degree to which K_eff,3axis_ preserves the rank ordering of K_eff,true_ (p is the probability of observing ρ ≥ this value by chance under the null hypothesis of no correlation). Across all 12 structures, ρ = 0.825 (p = 0.001); excluding the higher-order outlier mp-1104676 gives ρ = 0.827 (p = 0.002, n = 11), confirming that the result is not driven by a single structure. The rank correlation demonstrates that the relative ordering of K values is well preserved (Fig. 3(b)), validating the screening utility of the three-axis protocol for monoclinic systems.

**Fig. S2. E(θ) angular profiles in the ac-plane for 12 monoclinic structures (sorted by K_eff,3axis_ in descending order). Blue circles: DFT noncollinear SOC energies at θ = 20°, 40°, ..., 160°; red curve: sin²(θ − θ_min_) fit; green dashed line: b-axis energy E_b_ (not in ac-plane, shown for reference). Panel titles show K_eff,3axis_ and K_eff,true_ (MJ/m³). Eleven of twelve profiles are well described by the sin² model; mp-1104676 (bottom row, left) shows a double-minimum profile characteristic of higher-order anisotropy.**

## **2.3. Triclinic Systems (P1 / P¯1)**

Triclinic crystals (space groups P1 and P¯1) have no rotational or mirror symmetry elements. As a result, there are no symmetry constraints on the anisotropy tensor, and the easy-axis direction can point anywhere on the unit sphere. The three-axis protocol is therefore an approximation with no symmetry justification.

To assess the reliability of K_eff,3axis_ for triclinic structures, we performed additional SOC calculations along 16 Fibonacci-lattice sphere-sampled directions (uniformly distributed over the hemisphere z > 0) in addition to the three crystallographic axes, giving 19 total directions. Because the triclinic anisotropy tensor K has no symmetry constraints, we fit K directly from these 19 measurements. The second-order magnetocrystalline anisotropy energy is E(ŝ)/V = ŝᵀ K ŝ = Σ_ij_ K_ij_ s_i_ s_j_, where K is a real symmetric 3×3 matrix with 6 independent elements (K_aa_, K_bb_, K_cc_, K_ab_, K_ac_, K_bc_, with K_ij_ = K_ji_ imposed by time-reversal symmetry: E(ŝ) = E(−ŝ) eliminates all odd-order terms). For each of the N = 19 measured directions ŝ_α_ = (s_α_a_, s_α_b_, s_α_c_), the energy equation is linear in the 6 tensor elements plus a constant E_0_^11^:

E_α_ = K_aa_ s_α_a_² + K_bb_ s_α_b_² + K_cc_ s_α_c_² + 2K_ab_ s_α_a_ s_α_b_ + 2K_ac_ s_α_a_ s_α_c_ + 2K_bc_ s_α_b_ s_α_c_ + E_0_

(α = 1, …, 19)	(S22)

This overdetermined linear system (N = 19 equations, 7 unknowns) is solved by ordinary least squares. The fitted K is then diagonalized to yield eigenvalues λ_1_ ≤ λ_2_ ≤ λ_3_, giving the anisotropy barrier obtained from the difference between the saddle point and the minimum point consistent with the orthorhombic and monoclinic definitions:

K_eff,true_= (λ_2_ − λ_1_) / V	(S23)

Figure 3(c) compares K_eff,3axis_ and K_eff,true_ for 8 triclinic structures (mean tensor-fit R² = 0.883 across all 8 structures; three structures with R² < 0.90 (mp-3530, mp-1203999, mp-541510) may have significant higher-order anisotropy). The Spearman rank correlation is ρ = 0.571 (p = 0.139, n = 8) not statistically significant at n = 8. The absence of significant rank correlation reflects the fundamental limitation that the second-order approximation and sparse 19-direction sampling cannot fully capture the energy landscape of triclinic systems. We note that triclinic structures represent only 6.6% of the unlabeled candidate pool and 1.4% of the labeled training set, limiting their overall impact on the screening outcomes. For any high-priority triclinic candidates identified by the workflow, we recommend full-sphere SOC sampling to obtain a reliable K_eff_ estimate.

**3. Details of Candidate scope and sampling strategy**

To build an MCA-labeled dataset that matches the domain where the predictor will be deployed, we drew candidates from the MP (v2024.3) using three filters: structures must have an experimental synthesis report (ICSD) tag, be ferromagnetic at their reported ground state^12^, and have a formation energy below 0.1 eV per atom above the convex hull. This initial filtering yielded 9,320 candidates. At this stage we deliberately did not exclude heavy rare-earth elements (Eu-Lu) and actinide compositions: the training objective was to learn a general, symmetry-aware MCA mapping with sufficient dynamic range (anchored by the high-$K_{eff}$ regime often realized in heavy rare-earth and actinide systems), whereas elemental-availability constraints are imposed only at the screening stage.

To select a representative subset for labeling, we embedded each structure using a pretrained CGCNN and extracted the 32-dimensional convolutional features as structural encodings. The embedding distribution is visualized in Fig. S3 with t-SNE visualization. We partitioned the embedding space into six clusters and then performed farthest-point sampling within each cluster based on Euclidean distance to maximize coverage of the design space. This procedure selected 821 non-cubic structures for first-principles MCA labeling. In addition, 914 cubic structures from the candidate pool were assigned an $K_{eff}$ of zero, a direct result of our axis-aligned calculation protocol.

To further enrich the dataset, we incorporated 1,027 uniaxial structures from the Novamag database. We removed duplicate crystals using standard structural equivalence checks based on lattice, symmetry, and atomic positions. For overlapping structures, MCA values agreed, preserving qualitative scale and ranking. This resulted in a final labeled dataset of 2,762 structures in total which was used to train the MCA predictors.

To assess consistency with the Novamag database, we identified duplicate crystals between the two sources using pymatgen. The matching was performed on relaxed CIF structures, comparing lattice parameters, symmetry, and atomic positions under typical tolerances (lattice length and site tolerances of 0.2 and 0.3, and an angular tolerance of 5°), with primitive-cell and scaling options enabled to ensure equivalence across conventional settings.

Fig. S3. t-SNE visualization of CGCNN embeddings for all structures in the search space, with clusters indicated by color. Representative structures were selected from each cluster via farthest sampling for subsequent calculations.

For each matched pair, MCA values were compared in both energy per cell (μeV/cell) and energy density (MJ/m^3^), using

$K\text{ }[\mathrm{MJ}\text{/}m^{3}]\text{ }=\text{ }\frac{E_{\mathrm{MCA}}\text{ }[eV/cell]}{V\text{ }[{\overset{˚}{\text{A}}}^{3}/cell]}\times160,217.66$	(S1)

Across the three duplicate pairs (MnAu, FePd, Fe_2_P), the MCA sign and qualitative ranking are preserved. Absolute differences are ≤ 0.63 MJ/m³, which correspond to ≈ 52~335 μeV per cell when mapped using our relaxed volumes. At this scale, offsets are readily explained by minor variations in relaxed geometries and common numerical settings (e.g., k-point meshes, smearing, PAW datasets). Given this level of concordance, it is reasonable to treat the two sources as compatible and to merge them for model training. Pairwise values are summarized in Table S1.

Table S1. Pairwise comparison of overlapping entries between this work and the Novamag database.

| **ID** | **formula** | $\boldsymbol{E}_{\boldsymbol{MCA}}\boldsymbol{ours}$  $\boldsymbol{(\mu eV/cell)}$ | $\boldsymbol{K}_{\boldsymbol{1}}\boldsymbol{ours}$  $\boldsymbol{(}\boldsymbol{MJ}/{\boldsymbol{m}^{\boldsymbol{3}}}\boldsymbol{)}$ | $\boldsymbol{K}_{\boldsymbol{1}}\boldsymbol{Novamag}$  $\boldsymbol{(}\boldsymbol{MJ}/{\boldsymbol{m}^{\boldsymbol{3}}}\boldsymbol{)}$ | ${\boldsymbol{\Delta}\boldsymbol{K}}_{\boldsymbol{1}}$  $\boldsymbol{(}\boldsymbol{MJ}/{\boldsymbol{m}^{\boldsymbol{3}}}\boldsymbol{)}$ | $\boldsymbol{implied}\boldsymbol{\Delta E}_{\boldsymbol{MCA}}$  $\boldsymbol{(\mu eV/cell)}$ |
| --- | --- | --- | --- | --- | --- | --- |
| mp-12675 | MnAu | 153 | 0.727 | 0.101 | 0.627 | 131.8 |
| mp-2831 | FePd | 264 | 1.516 | 1.219 | 0.297 | 51.7 |
| mp-778 | Fe_2_P | 2392 | 3.815 | 3.281 | 0.534 | 334.6 |

**4. Details of feature engineering**

To represent the diverse chemical and structural characteristics of the candidate materials, we engineered a comprehensive set of 306 descriptors, which can be grouped into three categories. First, four stoichiometric descriptors, derived from the normalized stoichiometric coefficients (e.g., L2 norm, entropy), were used to quantify compositional complexity irrespective of element identity. Second, a rich set of 290 compositional descriptors was generated. These include two features representing elemental fraction and inclusion as one-hot vectors, with the vector space restricted to d- and f-block metals to focus on magnetically relevant elements. This was supplemented by 288 features derived by applying 12 statistical operations (e.g., mean, variance, maximum) to 24 fundamental properties of the constituent elements. Finally, 12 structural descriptors encoded geometric and topological information, ranging from basic lattice properties (e.g., cell volume, space group, lattice parameters) to advanced representations such as the eigenvalues of the Sine Matrix^13^ implemented in DScribe^14^ module and learned 32-dimensional embeddings from a pretrained CGCNN (see the SI for a detailed list of the features used). Random-forest^15^ classifiers and regressors from scikit-learn^16^ were then employed for all prediction tasks.

| **Feature type** | **Feature** | **Operation** |
| --- | --- | --- |
| Stoichiometric | Species number |  |
|  | L2 norm of atomic fraction |  |
|  | L3 norm of atomic fraction |  |
|  | Entropy of atomic fraction |  |
| Compositional | TM & RE fraction (no operation) | Average |
|  | TM & RE inclusion (no operation) | Maximum |
|  | Atomic number | Minimum |
|  | Mendeleev number | Maximum difference |
|  | Group number | Minimum difference |
|  | Period number | Standard deviation |
|  | Atomic volume | Weighted average |
|  | Spin only magnetic moment | Weighted maximum |
|  | Atomic mass | Weighted minimum |
|  | Electronegativity | Weighted maximum difference |
|  | Atomic radius | Weighted minimum difference |
|  | Ionization energy | Weighted standard deviation |
|  | Electron affinity |  |
|  | Dipole polarizability |  |
|  | Density |  |
|  | Boiling point |  |
|  | Melting point |  |
|  | Valence s electrons |  |
|  | Valence p electrons |  |
|  | Valence d electrons |  |
|  | Valence f electrons |  |
|  | Unfilled s electrons |  |
|  | Unfilled p electrons |  |
|  | Unfilled d electrons |  |
|  | Unfilled f electrons |  |
|  | Unpaired electrons |  |
| Structural | Crystal system |  |
|  | Space group number |  |
|  | Unit cell volume |  |
|  | Lattice constant mean ratio (arithmetic) |  |
|  | Lattice constant mean ratio (geometric) |  |
|  | Lattice angle mean ratio (arithmetic) |  |
|  | Lattice angle mean ratio (geometric) |  |
|  | Lattice constant coefficient of variation |  |
|  | Anisotropy factor |  |
|  | Lattice angle mean deviation |  |
|  | Sine matrix eigenvalues |  |
|  | CGCNN embedding |  |

Table S2. Summary of all features used for machine-learning analysis, categorized by feature type

A total of 12 structure-derived features were used to represent global and local geometric characteristics of each crystal. Basic crystallographic identifiers—the crystal system, space group number, and conventional cell volume—encode the underlying symmetry and scale of the lattice. To quantify lattice shape anisotropy, we defined both arithmetic and geometric lattice constant mean ratios,

$r_{\mathrm{arith}}=\frac{1}{3}\left( \frac{b}{a}+\frac{c}{a}+\frac{c}{b} \right)$	(S2)

$r_{\mathrm{geom}}=(b/a\cdot c/a\cdot c/b)^{1/3}$	(S3)

and analogous lattice angle mean ratios, obtained from the angles $(\alpha,\beta,\gamma)$ in the same arithmetic and geometric forms. These ratios approach unity for cubic or highly isotropic structures and increase as the lattice becomes distorted, thereby providing a direct quantitative measure of structural anisotropy. Further, three scalar descriptors capture the degree of deviation from perfect cubic symmetry: the coefficient of variation of the lattice constants,

$\mathrm{CV}=\frac{\mathrm{std}(a,b,c)}{\mathrm{mean}(a,b,c)}$	(S4)

the anisotropy factor,

$AF=\sqrt{\frac{1}{3}\sum_{i=a,b,c} {(\frac{x_{i}-\bar{x}}{\bar{x}})}^{2}}$	(S5)

and the mean angular deviation,

$\Delta_{\theta}=\frac{1}{3}(\mid\alpha-90\mid+\mid\beta-90\mid+\mid\gamma-90\mid)$	(S6).

All three increase monotonically with departure from cubic symmetry, serving as complementary indicators of long-range lattice distortion. Local and intermediate-range environments were encoded by sine matrix eigenvalues, which compactly represent atomic interactions modulated by interatomic distances, and by CGCNN embeddings, learned graph representations of the local atomic topology obtained from a pretrained crystal graph convolutional neural network. Together, these 12 descriptors encompass both global lattice geometry and local coordination information, forming a physically interpretable and numerically continuous feature space for machine-learning analysis of MCA.

**5. Details of fine-tuning MatterGen and evaluation of generated structures**

We fine-tuned a state-of-the-art MatterGen diffusion model, which was pretrained on a dataset of approximately 670,000 crystals labeled with magnetic density reported in $\mu_{B}/Å^{3} ADDIN ZOTERO\_ITEM CSL\_CITATION \{"citationID":"YHZAWG34","properties":\{"unsorted":false,"formattedCitation":"\backslash\backslash super 20\backslash\backslash nosupersub\{\}","plainCitation":"20","noteIndex":0\},"citationItems":[\{"id":33,"uris":["http://zotero.org/users/17444553/items/PV7QETFV"],"itemData":\{"id":33,"type":"article-journal","abstract":"The design of functional materials with desired properties is essential in driving technological advances in areas such as energy storage, catalysis and carbon capture1-3. Generative models accelerate materials design by directly generating new materials given desired property constraints, but current methods have a low success rate in proposing stable crystals or can satisfy only a limited set of property constraints4-11. Here we present MatterGen, a model that generates stable, diverse inorganic materials across the periodic table and can further be fine-tuned to steer the generation towards a broad range of property constraints. Compared with previous generative models4,12, structures produced by MatterGen are more than twice as likely to be new and stable, and more than ten times closer to the local energy minimum. After fine-tuning, MatterGen successfully generates stable, new materials with desired chemistry, symmetry and mechanical, electronic and magnetic properties. As a proof of concept, we synthesize one of the generated structures and measure its property value to be within 20\% of our target. We believe that the quality of generated materials and the breadth of abilities of MatterGen represent an important advancement towards creating a foundational generative model for materials design.","container-title":"Nature","DOI":"10.1038/s41586-025-08628-5","ISSN":"1476-4687","issue":"8055","page":"624-632","title":"A generative model for inorganic materials design","volume":"639","author":[\{"family":"Zeni","given":"Claudio"\},\{"family":"Pinsler","given":"Robert"\},\{"family":"Zügner","given":"Daniel"\},\{"family":"Fowler","given":"Andrew"\},\{"family":"Horton","given":"Matthew"\},\{"family":"Fu","given":"Xiang"\},\{"family":"Wang","given":"Zilong"\},\{"family":"Shysheya","given":"Aliaksandra"\},\{"family":"Crabbé","given":"Jonathan"\},\{"family":"Ueda","given":"Shoko"\},\{"family":"Sordillo","given":"Roberto"\},\{"family":"Sun","given":"Lixin"\},\{"family":"Smith","given":"Jake"\},\{"family":"Nguyen","given":"Bichlien"\},\{"family":"Schulz","given":"Hannes"\},\{"family":"Lewis","given":"Sarah"\},\{"family":"Huang","given":"Chin-Wei"\},\{"family":"Lu","given":"Ziheng"\},\{"family":"Zhou","given":"Yichi"\},\{"family":"Yang","given":"Han"\},\{"family":"Hao","given":"Hongxia"\},\{"family":"Li","given":"Jielan"\},\{"family":"Yang","given":"Chunlei"\},\{"family":"Li","given":"Wenjie"\},\{"family":"Tomioka","given":"Ryota"\},\{"family":"Xie","given":"Tian"\}],"issued":\{"date-parts":[["2025",3]]\}\}\}],"schema":"https://github.com/citation-style-language/schema/raw/master/csl-citation.json"\}$^20^. To align with the screening metric, we convert magnetic density to field units via

$\mu_{0}M_{s} [T] = 11.6\times\rho_{m} [\mu_{B}/Å^{3}]$,

where $\rho_{m}$ is the cell magnetic moment per volume. This constant follows from

$\mu_{B}=9.274\times{10}^{-24} [A\cdot m^{2}]$, $1 \left[ Å^{3} \right]={10}^{-30}[m^{3}]$,

and $\mu_{0}=4\pi\times{10}^{-7} {[H}/{m]}=4\pi\times{10}^{-7} [{T\cdot m}/A$]:

$M_{s} \left[ A/m \right]=\rho_{m}\cdot\frac{9.274\times{10}^{-24}}{{10}^{-30}}=9.274\times{10}^{6}\times\rho_{m}$, hence

$\mu_{0}M_{s} \left[ T \right]=4\pi\times{10}^{-7}\times9.274\times{10}^{6}\times\rho_{m}=11.6\times\rho_{m} [T]$.

Low-symmetry systems (triclinic, monoclinic, and orthorhombic) were excluded from fine-tuning due to label ambiguity when restricted to a/b/c directions. This was achieved by conditioning the generator on the raw $K_{eff}$ values of uniaxial crystal structures, which preserves the crucial sign information, enabling the model to distinguish between easy-axis and easy-plane regimes. To ensure efficient training, MatterGen model adopted a lightweight adaptation strategy (LoRA^17^) where the pretrained diffusion backbone was kept frozen while only specific adaptation layers were updated.

For the evaluation, stability was defined by an energy above the convex hull ($E_{hull}$) below 0.1 eV/atom. Uniqueness was ensured by identifying and removing duplicate structures within the generated set. Novelty was assessed by checking for prior existence in a combined MP and Alexandria database^18,19^ with ≤20 atom structures (alex_mp_20). The effect of this anisotropy conditioning on the sampled distribution is shown in Fig. S4.

Fig. S4. Effect of anisotropy fine-tuning on generative sampling. Distribution of ML-predicted effective anisotropy K_eff_ for structures sampled by the magnetization-only conditioned model (control; median 0.10 MJ/m³) and by the model additionally fine-tuned on magnetocrystalline anisotropy (test; median 0.16 MJ/m³). The anisotropy conditioning shifts the sampled distribution toward higher K_eff_ (one-sided Mann–Whitney U, p = 1.5×10⁻⁴), confirming that the fine-tuning biases generation toward harder magnets.

**6. Details of Easy-Axis Classification model performance**

Fig. S5. Precision-Recall (PR) curve for the easy-axis classifier. The curve illustrates the performance of the Random Forest model on the uniaxial dataset (n=1323). The selected operating point (red marker) is the one that maximizes the F1 score (0.678), corresponding to a decision threshold of τ = 0.302.

To establish a principled threshold, we analyzed the Precision-Recall (PR) curve derived from 5-fold out-of-fold predictions (Fig. S5). We selected the operating point that maximizes the F1 score, a standard metric for balancing precision and recall. This F1-optimal point corresponds to a threshold of τ = 0.302, which yields an exceptionally high recall of 0.978. The precision at this threshold is 0.519, which is necessarily close to the positive class prior of 0.51 (51:49 class balance), indicating that the classifier primarily acts to remove the most confidently predicted easy-plane candidates.

**7. Detailed list of DFT validated hard-magnet candidates**

**Fig. S6. Effective anisotropy K_eff_ versus saturation magnetization μ_0_M_s_ for the DFT-labeled structures used to train the regressor. Blue and red points denote rare-earth-free/-lean and rare-earth-containing compositions, respectively; solid, dashed and dotted green lines mark κ = 1, 0.5 and 0.1, and the shaded region (κ > 1) is the hard-magnet regime. Black stars are the benchmark magnets Nd₂Fe₁₄B (mp-5182) and Fe₁₆N₂ (mp-555). Twelve rare-earth-free or -lean structures already satisfy the magnetization and κ > 1 criteria and seed the candidate list of Table S3.**

Of the 33 MP-derived hard-magnet candidates tabulated below, 6 occupy low-symmetry crystal classes — 3 orthorhombic (mp-1077988, mp-19218, mp-22632) and 3 monoclinic (mp-29770, mp-568349, mp-1105304). They extend the sparse set of non-uniaxial κ > 1 hard-magnet candidates and are discussed in Section 3.3.

Table S3. MP-pool candidates retained by the screening and their key properties.
List of 33 MP entries meeting the selection criteria (Rare-earth-lean/-free status, μ_0_M_s_ > 0.5 T, κ > 1). Columns: Rare-earth-lean/-free, crystal system, identifier, composition, magnetization (T), and effective magnetic anisotropy coefficient (MJ/m³). Of these, 12 arose from the pre-labeled set used to train the model, and 21 were discovered by applying the trained model to previously unlabeled MP structures.

| **Rare-Earth inclusion** | **Crystal** | **ID** | **Composition** | **M_s_ (T)** | **K_eff_ (MJ/m^3^)** |
| --- | --- | --- | --- | --- | --- |
|  | Tetragonal | mp-1078664 | Pr_4_Mn_4_Se_4_O_6_ | 0.702 | 3.21 |
|  |  | mp-1079573 | Pr_4_Fe_4_S_4_O_6_ | 0.623 | 74.2 |
|  |  | mp-1080777 | Pr_4_Fe_4_Se_4_O_6_ | 0.582 | 5.03 |
|  |  | mp-19274 | BaPrMn_2_O_6_ | 0.660 | 4.03 |
|  |  | mp-20926 | BaNdCo_2_O_5_ | 0.559 | 2.38 |
|  |  | mp-22751 | BaPrCo_2_O_6_ | 0.605 | 1.81 |
|  |  | mp-1078182 | Nd_4_Fe_4_Se_4_O_6_ | 0.590 | 0.44 |
|  |  | mp-546665 | BaNdMn_2_O_5_ | 0.817 | 6.33 |
|  |  | mp-549776 | BaPrMn_2_O_5_ | 0.811 | 6.37 |
|  |  | mp-638374 | BaNdFe_2_O_5_ | 0.832 | 58.4 |
| No Rare-Earth | Monoclinic | mp-568349 | Cr_2_Fe_4_Se_8_ | 0.684 | 0.88 |
|  |  | mp-29770 | Na_6_Mn_8_Te_12_ | 0.539 | 1.47 |
|  |  | mp-1105304 | Ca_2_FeOsO_6_ | 0.765 | 0.94 |
|  | Orthorhombic | mp-1077988 | Mn_4_B_8_W_2_ | 0.677 | 1.06 |
|  |  | mp-19218 | Sr_6_Fe_4_O_10_ | 0.587 | 0.56 |
|  |  | mp-22632 | Co_6_B_4_O_12_ | 1.014 | 0.91 |
|  | Tetragonal | mp-1001836 | MnGa | 1.227 | 2.32 |
|  |  | mp-3702 | FeCuPt_2_ | 0.898 | 5.22 |
|  |  | mp-12565 | Mn_2_Au_8_ | 0.596 | 0.67 |
|  |  | mp-13463 | FeNiPt_2_ | 1.117 | 1.30 |
|  |  | mp-3021 | Tl_2_Fe_4_Se_4_ | 0.636 | 0.77 |
|  |  | mp-30411 | Mn_9_Au_31_ | 0.664 | 1.30 |
|  |  | mp-2260 | FePt | 1.481 | 16.3 |
|  |  | mp-771 | MnAl | 1.171 | 1.78 |
|  |  | mp-949 | CoPt | 1.051 | 6.54 |
|  |  | mp-18857 | Sr_4_Fe_2_Mo_2_O_12_ | 0.546 | 0.26 |
|  |  | mp-21118 | Sr_2_Mn_4_Ge_4_ | 0.538 | 2.28 |
|  |  | mp-23229 | Fe_3_Cl_6_ | 0.711 | 0.41 |
|  | Hexagonal | mp-374 | CrB_2_ | 0.510 | 0.90 |
|  |  | mp-29076 | Fe_2_W_2_N_4_ | 1.061 | 2.74 |
|  |  | mp-22130 | TiFe_6_Ge_6_ | 0.724 | 1.72 |
|  |  | mp-569151 | Mn_2_Ga_2_Pt_2_ | 1.037 | 3.08 |
|  |  | mp-20473 | Mn_4_Ge_2_ | 1.365 | 1.77 |

Table S4. Generated-pool hard-magnet candidates retained after DFT relaxation and spin–orbit validation. Five rare-earth-free structures with κ_DFT_ > 1. Columns: novelty status (novel, or matched Materials Project entry), crystal system, composition, magnetization (T), effective magnetic anisotropy (MJ/m³), and hardness parameter κ. All five are metastable ordered intermetallics (E_hull_ = 35–160 meV/atom); the three lead candidates Fe₂PtRh, the I4/mmm FeCoPt₂ (E_hull_ = 35 meV/atom), and TaFe₃ are confirmed dynamically stable by phonon calculations (Fig. S8).

| **MP entry status** | **Crystal system** | **Composition** | **M_s_ (T)** | **K_eff_ (MJ/m³)** | **κ** |
| --- | --- | --- | --- | --- | --- |
| Novel | Tetragonal | Fe₂PtRh | 1.43 | 15.1 | 3.04 |
| Novel | Tetragonal | MnCoPt₂ | 1.17 | 14.6 | 3.67 |
| Reported  (mp-1224993) | Tetragonal | FeCoPt₂ | 1.17 | 12.9 | 3.44 |
| Novel | Tetragonal | FeCoPt₂ | 1.18 | 8.2 | 2.72 |
| Novel | Trigonal | TaFe₃ | 1.02 | 3.8 | 2.15 |

**8. Details of Structural and Stability Analysis for Generated hard-magnet Candidates**

Fig. S7. Generated hard-magnet candidates. The five rare-earth-free structures retained after DFT relaxation and spin–orbit validation, shown as supercells. Labels give composition, space group, DFT formation energy above the convex hull, and novelty status (green = novel; grey = matches the reported Materials Project entry mp-1224993).

Fig. S8. Phonon band structures of the three lead generated candidates—Fe₂PtRh, the I4/mmm FeCoPt₂ ordering, and TaFe₃. All three are dynamically stable, with the lowest band minima within numerical noise of zero (≈ −0.01 to −0.02 THz).

**9. κ Screening Buffer and Threshold Sensitivity Analysis**

The screening pipeline applies ML-predicted κ (κ_ML_) as a buffer criterion before computationally expensive DFT validation: κ_ML_ > 0.8 for MP-derived candidates (where DFT-calculated crystal structure and M_s_ are available from the MP API) and κ_ML_ > 0.5 for MatterGen-generated structures (where M_s_ is estimated by CHGNet). The more conservative buffer for generated structures reflects the additional uncertainty from CHGNet M_s_ predictions. Table S5 shows the buffer was not restrictive for the final set: all five DFT-validated generated candidates had κ_ML_ > 1.0—retained even under the stricter κ_ML_ > 0.8 MP buffer—while their κ_DFT_ reached 2.2–3.7, so no final candidate lay near the buffer boundary.

**Table S5. Buffer sensitivity for the DFT-validated MatterGen candidates.**

| **MP entry** | **Formula** | **κ_ML_** | **κ_DFT_** | **Capt. at ≥0.5?** | **Capt. at ≥0.8?** | **Capt. at ≥1.0?** |
| --- | --- | --- | --- | --- | --- | --- |
| Novel | Fe₂PtRh | 1.03 | 3.04 | Yes | Yes | Yes |
| Novel | MnCoPt₂ | 1.01 | 3.67 | Yes | Yes | Yes |
| Reported (mp-1224993) | FeCoPt₂ | 1.37 | 3.44 | Yes | Yes | Yes |
| Novel | FeCoPt₂ | 1.12 | 2.72 | Yes | Yes | Yes |
| Novel | TaFe₃ | 1.47 | 2.15 | Yes | Yes | Yes |

Table S6 summarizes the sensitivity of the κ > 1 candidate count to the final DFT-calculated κ_DFT_ threshold. The yield is robust: 17 of the 21 DFT-validated MP candidates and all five generated candidates retain κ_DFT_ > 1.2, demonstrating that the main conclusions are insensitive to moderate tightening of the hard-magnet criterion.

**Table S6. κ_DFT_ threshold sensitivity (DFT-validated candidates).**

| **κ threshold** | **MP DFT-validated (n=73)** | **Generated DFT-validated (n=9)** |
| --- | --- | --- |
| κ > 0.8 | 23 | 5 |
| κ > 1.0 (current) | 21 | 5 |
| κ > 1.2 | 17 | 5 |

**10. Classification Threshold (τ) Sensitivity Analysis**

The binary classifier outputs a probability score for easy-axis anisotropy; predictions are made positive (easy-axis) when this score exceeds a threshold τ. The value τ = 0.302 was selected by maximizing F1 on the precision–recall curve (Sec S5) from 5-fold stratified cross-validation (N = 1,323 labeled structures, Random Forest, 500 trees). This threshold prioritizes recall to minimize missed candidates, accepting higher false-positive rates that are subsequently reduced by the regression and κ > 1.0 filter stages. Table S7 compares key metrics across a range of thresholds.

**Table S7. Classification threshold sensitivity (5-fold OOF cross-validation).**

| **τ** | **Precision** | **Recall** | **F1** | **TP** | **FP** | **FN** | **Miss rate** |
| --- | --- | --- | --- | --- | --- | --- | --- |
| 0.1 | 0.506 | 1.000 | 0.672 | 670 | 653 | 0 | 0.0% |
| 0.2 | 0.509 | 0.997 | 0.674 | 668 | 645 | 2 | 0.3% |
| 0.302 (★) | 0.517 | 0.964 | 0.673 | 646 | 604 | 24 | 3.6% |
| 0.4 | 0.535 | 0.836 | 0.653 | 560 | 486 | 110 | 16.4% |
| 0.5 | 0.565 | 0.570 | 0.568 | 382 | 294 | 288 | 43.0% |
| 0.7 | 0.683 | 0.084 | 0.149 | 56 | 26 | 614 | 91.6% |

★ Selected threshold. Miss rate = FN / (TP + FN).

False positives passed to the regression stage are subsequently filtered by the K_eff_ regression and κ > 1.0 criterion; only structures passing both stages advance to DFT validation, limiting the DFT cost impact of classification FPs.

**11. Feature Group Importance**

The Random Forest classification model uses all 632 features derived from the 306 descriptors described in Section 3 (Table S2), grouped here into five categories for importance analysis (Table S8). Feature importance was assessed as the mean impurity decrease averaged over 5-fold cross-validation (500 trees per fold). Note that the 306 descriptor count reflects conceptual descriptor types (e.g., the sine matrix and the CGCNN embedding are each counted as one descriptor), whereas the 632 feature count enumerates individual numeric columns in the input matrix (e.g., 192 sine-matrix eigenvalues and 32 CGCNN dimensions). Although elemental/compositional features dominate by total count (392 features, 74.1% cumulative importance), CGCNN graph embeddings contribute 16.9% despite comprising only 32 features — a per-feature importance 2.8× higher than compositional features. This reflects their ability to encode local atomic environment and coordination geometry information complementary to the global compositional descriptors. The top individual features (Fig. S9) are dominated by CGCNN embedding dimensions interleaved with structural features (cell volume ratio, anisotropy factor, lattice ratios), consistent with the physical intuition that crystal geometry and local bonding environment govern magnetic anisotropy.

The same analysis applied to the regression model yields a markedly different pattern (Table S9, Fig. S10): crystal system (50.5%) and structural/lattice features (31.4%) together account for more than 80% of total importance, while elemental/compositional features contribute only 15.3%. This pattern reflects the physical structure of the regression target: K_eff_ is identically zero for all cubic structures by symmetry, so the crystal-system encoding is the single most decisive predictor, and structural geometry (lattice ratios, cell volume, anisotropy factor) determines the magnitude of K_eff_ among non-cubic systems. In contrast, the classifier must identify element combinations that favor uniaxial over planar anisotropy, which explains why elemental/compositional features dominate there. CGCNN embeddings contribute 2.2% with a per-feature importance 1.7× higher than elemental/compositional features, consistent with their role in encoding local coordination geometry complementary to the global structural descriptors.

**Table S8. Feature group importance in the classification model.**

| **Feature group** | **N features** | **Cumulative importance** | **Per-feature importance** |
| --- | --- | --- | --- |
| Elemental/Compositional | 392 | 74.1% | 1.89 × 10⁻³ |
| CGCNN Embeddings | 32 | 16.9% | 5.29 × 10⁻³ (2.8× higher) |
| Sine-Matrix Eigenvalues | 192 | 4.7% | 0.24 × 10⁻³ |
| Structural/Lattice | 9 | 4.2% | — |
|  |  |  |  |
| Crystal System | 7 | 0.2% | — |

**Table S9. Feature group importance in the regression model.**

| **Feature group** | **N features** | **Cumulative importance** | **Per-feature importance** |
| --- | --- | --- | --- |
| Elemental/Compositional | 392 | 15.3% | 3.91 × 10⁻³ |
| CGCNN Embeddings | 32 | 2.2% | 6.77 × 10⁻⁴ (1.7× higher) |
| Sine-Matrix Eigenvalues | 192 | 0.7% | 3.43 × 10⁻⁵ |
| Structural/Lattice | 9 | 31.4% | — |
| Crystal System | 7 | 50.5% | — |

**Fig. S9. Feature group importance bar chart (RF classification, 5-fold CV). Error bars represent standard deviation across folds.**

**Fig. S10. Feature group importance bar chart (RF regression, 5-fold CV). Error bars represent standard deviation across folds.**

**12. DFT M_s_ Uncertainty Propagation to the Hardness Parameter κ**

Uncertainties arise at multiple stages of the screening pipeline—DFT errors in K_eff_ (sub-meV per cell), ML regression scatter (main-text Fig. 3a), and a systematic ~0.1–0.2 T offset between DFT-computed and experimental M_s_—but their combined effect on κ is most consequential only for borderline candidates near κ ≈ 1. The mandatory DFT validation step, applied to all screened candidates, provides an independent check that limits the propagation of these errors into false confirmations. Below we quantify the dominant M_s_ contribution.

The hardness parameter κ = √(K_eff_/μ_0_M_s_^2^) depends on both K_eff_ (from DFT MCA calculations) and M_s_ (from DFT magnetic moments). DFT systematically misestimates M_s_ by approximately 0.1–0.2 T relative to experimental values, primarily due to self-interaction errors and the neglect of orbital magnetic moments. Because κ depends on M_s_ quadratically but on K_eff_ only linearly, a given relative error in M_s_ propagates into κ twice as strongly as the same relative error in K_eff_; the M_s_ uncertainty therefore dominates, and the values in Table S10 represent conservative upper bounds on the classification uncertainty. For a representative ΔM_s_ = 0.2 T offset:

**Table S10. κ uncertainty from DFT M_s_ overestimation.**

| **M_s,DFT_ (T)** | **ΔM_s_ (T)** | **Δκ/κ** | **Safe if κ_DFT_ >** |
| --- | --- | --- | --- |
| 0.8 | 0.2 | 25% | 1.33 |
| 1.0 | 0.2 | 20% | 1.25 |
| 1.2 | 0.2 | 17% | 1.20 |

"Safe if κ_DFT_ >" gives the threshold above which the hard-magnet classification (κ > 1.0) is unaffected even with maximum M_s_ uncertainty. Of the five DFT-validated generated candidates, all have κ_DFT_ > 1.25 (2.15–3.67) and are robustly classified as hard magnets, comfortably outside the M_s_-uncertainty margin.

**13. Compositional and structural novelty of the generative-model candidates relative to the training data and the MP-derived hard-magnet candidates**

To test whether the diffusion-model candidates merely replicate compositions or structures seen during training, we quantify the distance of each of the five generated candidates to three reference sets: (i) the 33 MP-derived hard-magnet candidates of Table S3, (ii) the 692-structure uniaxial dataset used to fine-tune the generative model (Section 5), and (iii) the full 2,762-structure MCA-labeled dataset underlying the screening models (Section 3). All three sets are evaluated with the same six metrics, three compositional and three structural; the nearest-neighbor statistics are summarized in Table S11.

1. Element-presence Jaccard distance d_J_(A, B) = 1 − |A ∩ B| / |A ∪ B|, evaluated on the sets of constituent elements of formulas A and B. This captures whether two compounds draw from the same element pool, agnostic to stoichiometry.

2. Atomic-fraction cosine distance d_cos_(v_A_, v_B_) = 1 − v_A_ · v_B_ / (||v_A_|| ||v_B_||), where v_A_ is a 104-dimensional vector indexed by atomic number whose entries are the atomic fractions of each element in A. This captures stoichiometric similarity.

3. Magpie-style elemental-property distance: each composition is described by a 14-dimensional vector of fraction-weighted means and fraction-weighted standard deviations of 7 elemental properties (electronegativity, atomic number, atomic mass, group number, period number, atomic radius, Mendeleev number). After Z-scoring across the MP33 ∪ GEN5 pool, Euclidean distance is computed.

4. Exact structural duplication: each candidate is compared with every reference structure of the same reduced composition using pymatgen's StructureMatcher (ltol = 0.2, stol = 0.3, angle_tol = 5°, ElementComparator). This detects replication of a training structure up to lattice strain and site tolerance.

5. Prototype-level structural identity: the same comparison with the species-agnostic FrameworkComparator, which tests whether two structures share the same lattice decoration irrespective of chemical species. This detects element-substitution copies of a known prototype.

6. Continuous structural distance: the Euclidean distance between CrystalNN site-statistics fingerprints (mean and standard deviation of 61 local-order parameters over all sites; 122 dimensions). For each candidate we report the distance to its nearest reference structure, d_NN_, together with the percentile of d_NN_ within the distribution of intra-set nearest-neighbor distances of the reference set itself—that is, how isolated the candidate would be if it were inserted into that set.

Applying these metrics to the five generated candidates against the 33 MP-derived candidates, the four Pt-bearing candidates—Fe₂PtRh, MnCoPt₂, and the two FeCoPt₂ orderings—sit close to the established L1₀ platinum magnets, with minimum element-Jaccard and atomic-fraction-cosine distances of 0.33 and 0.13 to FePt or CoPt; the generator is refining a known hard-magnet chemistry. TaFe₃ is the clear outlier, at Jaccard 0.67 and cosine 0.33 (nearest neighbor FePt), and is the only candidate whose composition is absent from the MP altogether—no MP hard magnet contains Ta. The generative model thus both densifies an established Pt-based region and reaches a rare-earth- and noble-metal-free composition family (Ta–Fe) used by no MP-derived hard magnet.

At the structure level, pymatgen's StructureMatcher confirms that only one candidate, the FeCoPt₂ ordering at E_hull_ = 63 meV/atom, reproduces a MP entry (mp-1224993, RMSD 0.008); the I4/mmm FeCoPt₂ ordering, Fe₂PtRh, MnCoPt₂, and TaFe₃ match no MP structure. The compositions Fe₂PtRh and MnCoPt₂ appear in the database only as recent machine-generated entries, and the relaxed candidates do not match even those, confirming they are distinct ordered phases.

The same conclusions hold, and sharpen, against the training data. None of the five candidates matches any structure in either training set under StructureMatcher. The four Pt-bearing candidates occur in neither training set at their exact compositions; their nearest structural neighbors are CuAu(L1₀)-type orderings and their layered derivatives—FePt and FeNiPt₂ within the MP-derived candidate set, and the isostructural prototypes NiPt, TiHg, and TiCdHg₂ within the training sets—at fingerprint distances within the bulk of the intra-set distribution (34th–83rd percentile across the two training sets; Table S11). Structurally as well as compositionally, these candidates are new element decorations of an established structural family—consistent with the local-refinement picture above.

TaFe₃ requires a more precise statement, which strengthens the novelty conclusion. The Ta–Fe system is not absent from the training corpus: the labeled dataset inherits 42 hypothetical binary Ta–Fe entries from the Novamag enumeration, including eleven TaFe₃ polymorphs carrying both easy-plane and easy-axis labels, and the fine-tuning set contains exactly one TaFe₃ structure—a tetragonal P4/mmm ordering labeled easy-plane (K_eff_ = −0.52 MJ/m³). None of these structures matches the generated candidate. The relaxed P3m1 phase—an ordered Fe-rich decoration of the C14 Laves framework of the equilibrium phase TaFe₂ (FrameworkComparator match)—matches no entry of either training set, and its fingerprint distance to the nearest training structure (d_NN_ = 0.35; nearest neighbor, the μ-phase Ta₆Fe₇) lies at the 90th percentile of the intra-set nearest-neighbor distribution: the generated structure is farther from every training structure than 90% of the training structures are from their own nearest neighbors. The only TaFe₃ structure the generative model saw during fine-tuning is an easy-plane phase, yet the model proposed a structurally distinct polymorph that DFT confirms as an easy-axis hard magnet (κ = 2.15; Fig. S11). Within an already-enumerated chemical system, the generative model therefore produced a previously unenumerated ordering with qualitatively different—and targeted—anisotropy, the opposite of training-set replication.

**Fig. S11. Polymorph-level novelty of the generated TaFe₃ candidate. (a) The only TaFe₃ structure seen during fine-tuning: a tetragonal P4/mmm ordering from the Novamag enumeration, labeled easy-plane (K_eff_ = −0.52 MJ/m³). (b) The generated trigonal P3m1 polymorph, an ordered Fe-rich decoration of the C14 Laves framework of TaFe₂, which DFT confirms as an easy-axis, phonon-stable hard magnet (K_eff_ = 3.8 MJ/m³, κ = 2.15). Fe and Ta atoms are shown in orange and blue, respectively.**

**Table S11. Nearest-neighbor novelty statistics of the five generated candidates against the three reference sets: the 33 MP-derived hard-magnet candidates (MP-33), the 692-structure fine-tuning set (FT-692), and the 2,762-structure MCA-labeled dataset (Corpus-2762). d_J_ and d_cos_ are the minimum element-Jaccard and atomic-fraction cosine distances (nearest composition in parentheses); exact match refers to StructureMatcher with ElementComparator; d_NN_ is the CrystalNN fingerprint distance to the nearest reference structure (in parentheses), with its percentile within the intra-set nearest-neighbor distance distribution of that reference set. The FeCoPt₂ ordering at E_hull_ = 63 meV/atom reproduces MP entry mp-1224993 (main text); that entry belongs to neither training set.**

| **Candidate** | **Reference set** | **d_J,min_** | **d_cos,min_ (nearest)** | **Exact match** | **d_NN_ (nearest)** | **d_NN_ percentile** |
| --- | --- | --- | --- | --- | --- | --- |
| Fe₂PtRh | MP-33 | 0.33 | 0.13 (FePt) | none | 0.09 (FePt) | 33rd |
|  | FT-692 | 0.33 | 0.09 (Fe₂Rh) | none | 0.03 (TiHg) | 37th |
|  | Corpus-2762 | 0.33 | 0.09 (Fe₂Pt) | none | 0.02 (NiPt) | 34th |
| MnCoPt₂ | MP-33 | 0.33 | 0.13 (CoPt) | none | 0.29 (FeNiPt₂) | 52nd |
|  | FT-692 | 0.33 | 0.09 (CoPt₂) | none | 0.22 (TiCdHg₂) | 81st |
|  | Corpus-2762 | 0.33 | 0.09 (CoPt₂) | none | 0.22 (TiCdHg₂) | 83rd |
| FeCoPt₂ (I4/mmm) | MP-33 | 0.33 | 0.13 (CoPt) | none | 0.09 (FeNiPt₂) | 33rd |
|  | FT-692 | 0.33 | 0.09 (CoPt₂) | none | 0.07 (FeNiPt₂) | 52nd |
|  | Corpus-2762 | 0.33 | 0.09 (CoPt₂) | none | 0.09 (FeNiPt₂) | 65th |
| FeCoPt₂ (mp-1224993) | MP-33 | 0.33 | 0.13 (CoPt) | none | 0.04 (FePt) | 12th |
|  | FT-692 | 0.33 | 0.09 (CoPt₂) | none | 0.03 (FePt) | 40th |
|  | Corpus-2762 | 0.33 | 0.09 (CoPt₂) | none | 0.03 (FePt) | 38th |
| TaFe₃ | MP-33 | 0.67 | 0.33 (FePt) | none | 0.73 (MnAu₄) | 79th |
|  | FT-692 | 0.00 | 0.00 (TaFe₃, P4/mmm) | none | 0.35 (Ta₆Fe₇) | 90th |
|  | Corpus-2762 | 0.00 | 0.00 (TaFe₃, hyp.) | none | 0.36 (Nb₆Co₇) | 90th |

**14. Screening Pipeline Details**

The screening pipeline (Fig. 2) was applied uniformly to the 9,320 experimentally reported MP ferromagnets and the 1,024 generated structures in four sequential stages. (i) Compositional filtering excluded heavy-rare-earth (Eu–Lu) and actinide-containing compositions. (ii) Magnetization screening used reported DFT moments for MP structures and CHGNet-predicted magnetization (an upper bound on M_s_) for generated structures, retaining entries with μ_0_M_s_ > 0.5 T. (iii) Easy-axis gating evaluated uniaxial structures with the binary classifier, forwarding only predicted easy-axis cases to regression; non-uniaxial structures were passed directly to regression. (iv) Hardness scoring applied the K_eff_ regressor and computed κ = √(K_eff_/μ_0_M_s_^2^). MP entries with μ_0_M_s_ > 0.5 T and κ_ML_ > 0.8 advanced to DFT validation; generated structures used a relaxed κ_ML_ > 0.5 buffer to absorb the additional uncertainty of their ML-estimated geometry (MatterSim) and magnetization (CHGNet). Generated structures additionally passed a MACE/SevenNet energy-above-hull consensus and, before DFT, explicit collinear magnetic-ordering enumeration: ferromagnetic, antiferromagnetic, and per-species ferrimagnetic orderings were generated with pymatgen's MagneticStructureEnumerator (symmetry-truncated, up to six orderings per structure with the ferromagnetic ordering retained), with magnetic sites and seed moments taken from CHGNet per-site predictions (|m| < 0.5 μB treated as non-magnetic); the lowest-DFT-energy ordering defines the ground state, guarding against collinear-ferromagnetic moments that collapse under a lower-energy antiferromagnetic ground state. Because permanent-magnet performance requires high magnetization, the DFT-intensive validation was focused on the high-moment candidates (μ_0_M_s_ ≥ 1 T).

**15. References**

(1)	Blöchl, P. E. Projector Augmented-Wave Method. *Phys. Rev. B* **1994**, *50* (24), 17953–17979. https://doi.org/10.1103/PhysRevB.50.17953.

(2)	Kresse, G.; Joubert, D. From Ultrasoft Pseudopotentials to the Projector Augmented-Wave Method. *Phys. Rev. B* **1999**, *59* (3), 1758–1775. https://doi.org/10.1103/PhysRevB.59.1758.

(3)	Perdew, J. P.; Burke, K.; Ernzerhof, M. Generalized Gradient Approximation Made Simple. *Phys. Rev. Lett.* **1996**, *77* (18), 3865–3868. https://doi.org/10.1103/PhysRevLett.77.3865.

(4)	Dudarev, S. L. Electron-Energy-Loss Spectra and the Structural Stability of Nickel Oxide: An LSDA+U Study. *Phys. Rev. B* **1998**, *57* (3), 1505–1509. https://doi.org/10.1103/PhysRevB.57.1505.

(5)	Locht, I. L. M.; Kvashnin, Y. O.; Rodrigues, D. C. M.; Pereiro, M.; Bergman, A.; Bergqvist, L.; Lichtenstein, A. I.; Katsnelson, M. I.; Delin, A.; Klautau, A. B.; Johansson, B.; Di Marco, I.; Eriksson, O. Standard Model of the Rare Earths Analyzed from the Hubbard I Approximation. *Phys. Rev. B* **2016**, *94* (8), 085137. https://doi.org/10.1103/PhysRevB.94.085137.

(6)	Kývala, L. Lattice Dynamics and Thermal Properties of Thorium Metal and Thorium Monocarbide. *Phys. Rev. B* **2020**, *101* (7), 075117. https://doi.org/10.1103/PhysRevB.101.075117.

(7)	Shields, A. E.; Santos-Carballal, D.; de Leeuw, N. H. A Density Functional Theory Study of Uranium-Doped Thoria and Uranium Adatoms on the Major Surfaces of Thorium Dioxide. *J. Nucl. Mater.* **2016**, *473*, 99–111. https://doi.org/10.1016/j.jnucmat.2016.02.009.

(8)	Obodo, K. O.; Chetty, N. First Principles LDA + U and GGA + U Study of Protactinium and Protactinium Oxides: Dependence on the Effective U Parameter. *J. Phys.: Condens. Matter* **2013**, *25* (14), 145603. https://doi.org/10.1088/0953-8984/25/14/145603.

(9)	Wen, X.-D.; Martin, R. L.; Henderson, T. M.; Scuseria, G. E. Density Functional Theory Studies of the Electronic Structure of Solid State Actinide Oxides. *Chem. Rev.* **2013**, *113* (2), 1063–1096. https://doi.org/10.1021/cr300374y.

(10)	Ong, S. P.; Richards, W. D.; Jain, A.; Hautier, G.; Kocher, M.; Cholia, S.; Gunter, D.; Chevrier, V. L.; Persson, K. A.; Ceder, G. Python Materials Genomics (Pymatgen): A Robust, Open-Source Python Library for Materials Analysis. *Comput. Mater. Sci.* **2013**, *68*, 314–319. https://doi.org/10.1016/j.commatsci.2012.10.028.

(11)	Skomski, R. Models of Magnetic Anisotropy. In *Simple Models of Magnetism*; Skomski, R., Ed.; Oxford University Press, 2008. https://doi.org/10.1093/acprof:oso/9780198570752.003.0003.

(12)	Horton, M. K.; Montoya, J. H.; Liu, M.; Persson, K. A. High-Throughput Prediction of the Ground-State Collinear Magnetic Order of Inorganic Materials Using Density Functional Theory. *Npj Comput. Mater.* **2019**, *5* (1), 64. https://doi.org/10.1038/s41524-019-0199-7.

(13)	Faber, F.; Lindmaa, A.; von Lilienfeld, O. A.; Armiento, R. Crystal Structure Representations for Machine Learning Models of Formation Energies. *Int. J. Quantum Chem.* **2015**, *115* (16), 1094–1101. https://doi.org/10.1002/qua.24917.

(14)	Himanen, L.; Jäger, M. O. J.; Morooka, E. V.; Federici Canova, F.; Ranawat, Y. S.; Gao, D. Z.; Rinke, P.; Foster, A. S. DScribe: Library of Descriptors for Machine Learning in Materials Science. *Comput. Phys. Commun.* **2020**, *247*, 106949. https://doi.org/10.1016/j.cpc.2019.106949.

(15)	Breiman, L. Random Forests. *Mach. Learn.* **2001**, *45* (1), 5–32. https://doi.org/10.1023/A:1010933404324.

(16)	Pedregosa, F.; Varoquaux, G.; Gramfort, A.; Michel, V.; Thirion, B.; Grisel, O.; Blondel, M.; Prettenhofer, P.; Weiss, R.; Dubourg, V.; Vanderplas, J.; Passos, A.; Cournapeau, D.; Brucher, M.; Perrot, M.; Duchesnay, É. Scikit-Learn: Machine Learning in Python. *J. Mach. Learn. Res.* **2011**, *12*, 2825–2830.

(17)	Hu, E. J.; Shen, Y.; Wallis, P.; Allen-Zhu, Z.; Li, Y.; Wang, S.; Wang, L.; Chen, W. LoRA: Low-Rank Adaptation of Large Language Models. arXiv October 16, 2021. https://doi.org/10.48550/arXiv.2106.09685.

(18)	Schmidt, J.; Hoffmann, N.; Wang, H.-C.; Borlido, P.; Carriço, P. J. M. A.; Cerqueira, T. F. T.; Botti, S.; Marques, M. A. L. Machine-Learning-Assisted Determination of the Global Zero-Temperature Phase Diagram of Materials. *Adv. Mater.* **2023**, *35* (22), 2210788. https://doi.org/10.1002/adma.202210788.

(19)	Schmidt, J.; Wang, H.-C.; Cerqueira, T. F. T.; Botti, S.; Marques, M. A. L. A Dataset of 175k Stable and Metastable Materials Calculated with the PBEsol and SCAN Functionals. *Sci. Data* **2022**, *9* (1), 64. https://doi.org/10.1038/s41597-022-01177-w.

(20)	Zeni, C.; Pinsler, R.; Zügner, D.; Fowler, A.; Horton, M.; Fu, X.; Wang, Z.; Shysheya, A.; Crabbé, J.; Ueda, S.; Sordillo, R.; Sun, L.; Smith, J.; Nguyen, B.; Schulz, H.; Lewis, S.; Huang, C.-W.; Lu, Z.; Zhou, Y.; Yang, H.; Hao, H.; Li, J.; Yang, C.; Li, W.; Tomioka, R.; Xie, T. A Generative Model for Inorganic Materials Design. *Nature* **2025**, *639* (8055), 624–632. https://doi.org/10.1038/s41586-025-08628-5.
